# Emerging role of exosomes in cancer progression and tumor microenvironment remodeling

**DOI:** 10.1186/s13045-022-01305-4

**Published:** 2022-06-28

**Authors:** Mahshid Deldar Abad Paskeh, Maliheh Entezari, Sepideh Mirzaei, Amirhossein Zabolian, Hossein Saleki, Mohamad Javad Naghdi, Sina Sabet, Mohammad Amin Khoshbakht, Mehrdad Hashemi, Kiavash Hushmandi, Gautam Sethi, Ali Zarrabi, Alan Prem Kumar, Shing Cheng Tan, Marios Papadakis, Athanasios Alexiou, Md Asiful Islam, Ebrahim Mostafavi, Milad Ashrafizadeh

**Affiliations:** 1 Department of Genetics, Faculty of Advanced Science and Technology, Tehran Medical Sciences, Islamic Azad University, Tehran, Iran; 2 Farhikhtegan Medical Convergence Sciences Research Center, Farhikhtegan Hospital Tehran Medical Sciences, Islamic Azad University, Tehran, Iran; 3 Department of Biology, Faculty of Science, Islamic Azad University, Science and Research Branch, Tehran, Iran; 4 Young Researchers and Elite Club, Tehran Medical Sciences, Islamic Azad University, Tehran, Iran; 5 Division of Epidemiology, Department of Food Hygiene and Quality Control, Faculty of Veterinary Medicine, University of Tehran, Tehran, Iran; 6 Department of Pharmacology, Yong Loo Lin School of Medicine, National University of Singapore, Singapore, 117600 Singapore; 7 NUS Centre for Cancer Research (N2CR), Yong Loo Lin School of Medicine, National University of Singapore, Singapore, 117597 Singapore; 8 Department of Biomedical Engineering, Faculty of Engineering and Natural Sciences, Istinye University, 34396 Istanbul, Turkey; 9 UKM Medical Molecular Biology Institute, Universiti Kebangsaan Malaysia, Kuala Lumpur, Malaysia; 10 Department of Surgery II, University Hospital Witten-Herdecke, University of Witten-Herdecke, Heusnerstrasse 40, 42283 Wuppertal, Germany; 11 Department of Science and Engineering, Novel Global Community Educational Foundation, Hebersham, Australia; 12 Department of Haematology, School of Medical Sciences, Universiti Sains Malaysia, Kubang Kerian, Kelantan Malaysia; 13 Stanford Cardiovascular Institute, Stanford University School of Medicine, Stanford, CA 94305 USA; 14 Department of Medicine, Stanford University School of Medicine, Stanford, CA 94305 USA; 15 Faculty of Engineering and Natural Sciences, Sabanci University, Orta Mahalle, Üniversite Caddesi No. 27, Orhanlı, Tuzla, Istanbul, Turkey; 16 AFNP Med Austria, Vienna, Austria; 17 Institute of Metabolism and Systems Research, University of Birmingham, Birmingham, B15 2TT UK

**Keywords:** Exosome, Cancer, Immunotherapy, Non-coding RNA, Biomarker, Prognosis

## Abstract

Cancer is one of the leading causes of death worldwide, and the factors responsible for its progression need to be elucidated. Exosomes are structures with an average size of 100 nm that can transport proteins, lipids, and nucleic acids. This review focuses on the role of exosomes in cancer progression and therapy. We discuss how exosomes are able to modulate components of the tumor microenvironment and influence proliferation and migration rates of cancer cells. We also highlight that, depending on their cargo, exosomes can suppress or promote tumor cell progression and can enhance or reduce cancer cell response to radio- and chemo-therapies. In addition, we describe how exosomes can trigger chronic inflammation and lead to immune evasion and tumor progression by focusing on their ability to transfer non-coding RNAs between cells and modulate other molecular signaling pathways such as PTEN and PI3K/Akt in cancer. Subsequently, we discuss the use of exosomes as carriers of anti-tumor agents and genetic tools to control cancer progression. We then discuss the role of tumor-derived exosomes in carcinogenesis. Finally, we devote a section to the study of exosomes as diagnostic and prognostic tools in clinical courses that is important for the treatment of cancer patients. This review provides a comprehensive understanding of the role of exosomes in cancer therapy, focusing on their therapeutic value in cancer progression and remodeling of the tumor microenvironment.

**Graphical Abstract:**

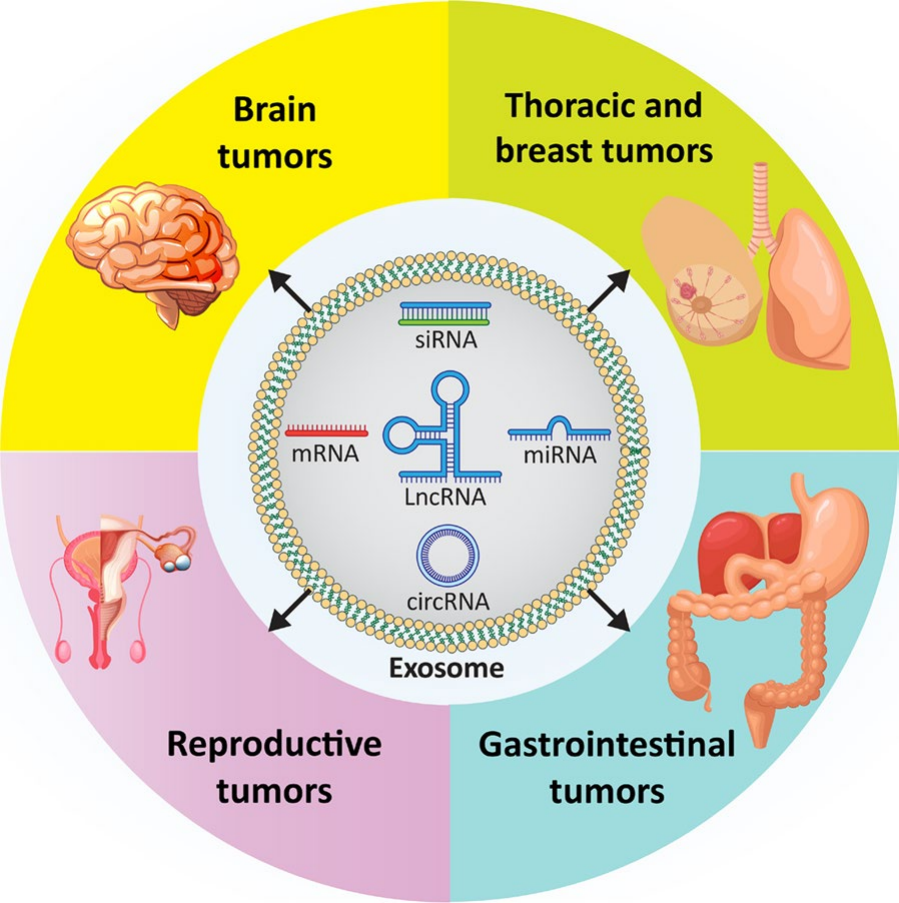

## Introduction

Cancer remains one of the diseases that threaten the lives of many people around the world. It is the second leading cause of death worldwide after cardiovascular diseases. Cancer cells possess unique features such as high proliferation rate, self-renewal ability, cancer stem cell (CSC) characteristics, metastasis, and the ability to switch between different molecular pathways to develop drug resistance [1–4]. Based on these properties, novel therapeutics, including nucleic acid drugs and anti-cancer agents, have been developed to target cancer cells and suppress their progression [5–9]. In addition, novel methods such as the use of nanoparticles have been employed for targeted delivery of therapeutics to cancer cells [10].

Recently, attention has focused on the role of a new type of structure, called extracellular vesicles (EVs) in cancer [11, 12]. EVs originate from the cell membrane and are considered micro- or nanovesicles. These structures can be secreted by all prokaryotic and eukaryotic cells in an evolutionarily conserved manner [13, 14]. Initially, EVs were thought to be waste products of cells or entities formed by cellular damage [15]. However, further studies on EVs have shown that they have vital biological functions and are important cellular components [16, 17]. There are several types of EVs that are categorized based on their size, origin, and localization [18–20]. The best known EVs include exosomes, microparticles, shedding vesicles, apoptotic bodies, tolerosomes, proteasomes, and prominosomes [21, 22]. There are two different mechanisms for the formation of EVs. In the first mechanism, EVs arise directly from cell membrane budding [15]. In the second mechanism, EVs arise during exocytosis of multivesicular bodies as part of the endocytosis system [23]. EVs are involved in biological functions in cells and play an important role in pathological conditions. They can transfer various molecules between cells and are a means of communication [24]. Therefore, special attention should be paid to their role in diseases, especially cancer [25–30].

The present review focuses on the role of exosomes in cancer. This comprehensive review first provides an overview of the discovery of exosomes, their composition, and the pathway of their biogenesis, which are of important for understanding these structures. Then, we focus specifically on the role of exosomes in cancer by introducing a section on exosomes in tumor microenvironment (TME) remodeling and how they influence various cancer hallmarks, including proliferation, migration, and therapy response. Next, we discuss exosomal non-coding RNAs (ncRNAs) and how they can affect cancer cell progression. We then turn our attention to exosomes and the key molecular signaling pathways that regulate cancer progression. Finally, we provide insight into tumor-derived exosomes and the clinical applications of exosomes relevant to the treatment of cancer patients.

## Exosome structure, isolation and dosing

Exosomes are double-membraned vesicles (30–150 nm in size; average particle size: 100 nm) secreted by different types of cells. Their specific functions depend on their origin. For example, exosomes originating from tumor cells provide cell-to-cell communication and are mainly involved in migration and invasion [31]. In the phospholipid membrane of exosomes originating from the parent cell, there are a variety of proteins and lipids [32, 33]. Among the lipid molecules, phosphatidylcholine, phosphatidylethanolamines, phosphatidylinositol, phosphatidylserine, and sphingomyelin are present in the exosome membrane. The composition and levels of these lipid molecules mainly influence the properties of exosomes. For example, the high stability of exosomes in body fluids and at different pH values is due to the high levels of sphingomyelin and phosphatidylinositol in their membrane. Therefore, these lipid molecules protect exosomes from degradation by proteolytic or lipolytic enzymes [34]. The phospholipid membrane of exosomes has lipid rafts containing proteins such as tyrosine kinase Src and glycosylphosphatidylinositol-containing proteins [35]. The presence of proteins in exosomes is a bit complex. Exosomes are thought to contain both general and specific proteins. The general or nonspecific proteins are present in all cell types, including CD63, tetraspanins, CD81, and CD9, whereas specific proteins include MHC II found in exosomes from dendritic cells and B lymphocytes, HER2 in exosomes from breast cancer, and EGFR in exosomes from gliomas [36]. It is worth noting that nonspecific proteins are critical for exosome function. Tetraspanins, for example, are nonspecific proteins that can interact with integrin or MHC molecules and form complexes. In addition to proteins, exosomes may also contain ncRNAs including microRNAs (miRNAs), long noncoding RNAs (lncRNAs) and circular RNAs (circRNAs) [37–39].

Since exosomes are present in various body fluids, they can be considered as novel biomarkers for the detection and diagnosis of various diseases. Therefore, it is important to develop methods for their isolation. A total of six strategies have been developed for the isolation of exosomes, including ultracentrifugation, ultrafiltration, size exclusion chromatography, precipitation, immunoaffinity-based capture, and microfluidics. Each method has its own advantages and problems that should be addressed [40–45]. Ultracentrifugation is capable of detecting exosomes based on their density, size, and shape, and its advantages include affordability, large sample capacity, and ability to isolate high concentrations of exosomes. The disadvantages of ultracentrifugation are the time-consuming process, the risk of exosome damage to exosomes from high-speed centrifugation, and the need for complex equipment [46–48]. Ultrafiltration isolates exosomes based on size differences from other particles. This strategy is fast and portable but has drawbacks such as low purity, shear stress, exosome loss, and clogging [49–51]. Size exclusion chromatography also uses size differences and has the advantage that it can accurately separate exosomes and isolate the intact exosomes without damaging them. Its disadvantage is the time-consuming process, which needs further advancement and development [52–54]. The precipitation method is based on changing the solubility of exosomes, and its advantages include the ease of performance, applicability to large sample volumes, and little damage to exosomes. Its disadvantages include the time-consuming process and the possibility of precipitating other particles such as polymeric materials and proteins [55, 56]. Immunoaffinity-based capture is based on the interaction between antibodies and antigens. Advantages of this method include high purity and the possibility of subtyping, whereas problems include high cost, low yield, risk of antigen blockade, and loss of exosome functionality [57]. The final technique for exosome isolation is the microfluidic strategy, which has the advantage of being inexpensive, time-saving, and requiring only a small amount of sample, but it has low sensitivity [58–62]. Further information on exosome isolation techniques has been reviewed elsewhere [63, 64].

The dosing of exosome has been the subject of debate and investigation in recent years. Three different methods have been used to determine exosome dosage, including cell equivalents, protein concentration, and/or specific quantitative analytical measurements using tools, with each with its own advantages and disadvantages. However, there is still a need to develop a standardized method for exosome dosing and currently available technologies suffer from accurate and precise assessment of exosomes at the level of individual vesicles. To improve the accuracy in exosome dosing, it is proposed to use multiple methods. For example, although the protein method that assesses total protein levels is fast and inexpensive, it may also assess proteins that are not exosome-related and may not indicate bioactive ingredients. TRPS, NTA, ELISA, cell equivalents, and flow cytometry are other methods for exosome dosing. A review by Willis and colleagues provides more details on techniques related to exosome dosing [65]. With regard to the use of exosomes in clinical trials, good manufacturing practices (GMPs) are important. Indeed, exosomes used in clinical trials should comply with GMPs. GMPs for exosomes consider three major factors, including upstream cell cultivation, downstream purification process, and exosome quality control [66].

## Biogenesis route of exosomes

Exosomes are formed by the endocytic pathway after passing through several steps [67]. In the first step, invaginations of the cytoplasmic membrane generate an early secretory endosome. Then, biogenesis of multivesicular bodies (MVBs) occurs by inward sprouting, generating intraluminal vesicles (ILVs) surrounded by endosomes. Acidification is then required for maturation of the late endosomes. In the final step, the ILVs fuse with the cell membrane and the exosomes are released [68]. MVBs have a size of 250–100 nm and therefore multiple ILVs with a particle size of 30–150 nm can be formed within the MVBs [69]. A number of proteins are involved in the formation of ILVs and MVBs, and in cargo selection [70]. The best known proteins for exosome biogenesis are the endosomal sorting complexes required for transport (ESCRT), which consist of four members, including ESCRT-0, -I, -II, and -III, that play a special role in membrane formation and cargo sorting [71]. Association of ubiquitylated cargoes with lipid microdomains is performed by ESCRT-0 and ESCRT-I. Then, ESCRT-II and -III are involved in invagination and formation of MVBs and ILVs. ALIX (Apoptosis-linked gene 2-interacting protein X, encoded by PDCD6IP), VTA1 (Vesicle Trafficking 1), VPS4 (Vacuolar protein sorting-associated protein 4), and TSG101 (Tumor susceptibility gene 101 protein) are other proteins that help the ESCRT machinery in exosome biogenesis.

Of note, there is another pathway for exosome biogenesis that is independent of ESCRT. In this ESCRT-independent pathway, heat shock protein-60 (HSP60), HSP70, and HSP90 act as chaperones, and CD63, CD81, CD82, CD37, and CD9 act as tetraspanins, which play important roles in membrane formation and cargo binding to lipid microdomains. Therefore, the mechanisms of exosome biogenesis are divided into two categories: ESCRT-dependent and -independent mechanisms [13, 72–75]. Among the tetraspanins, CD63 and CD81 are the most abundant proteins in the membranes of ILVs and are considered to be markers for exosomes [75, 76].

**Fig. 1:**
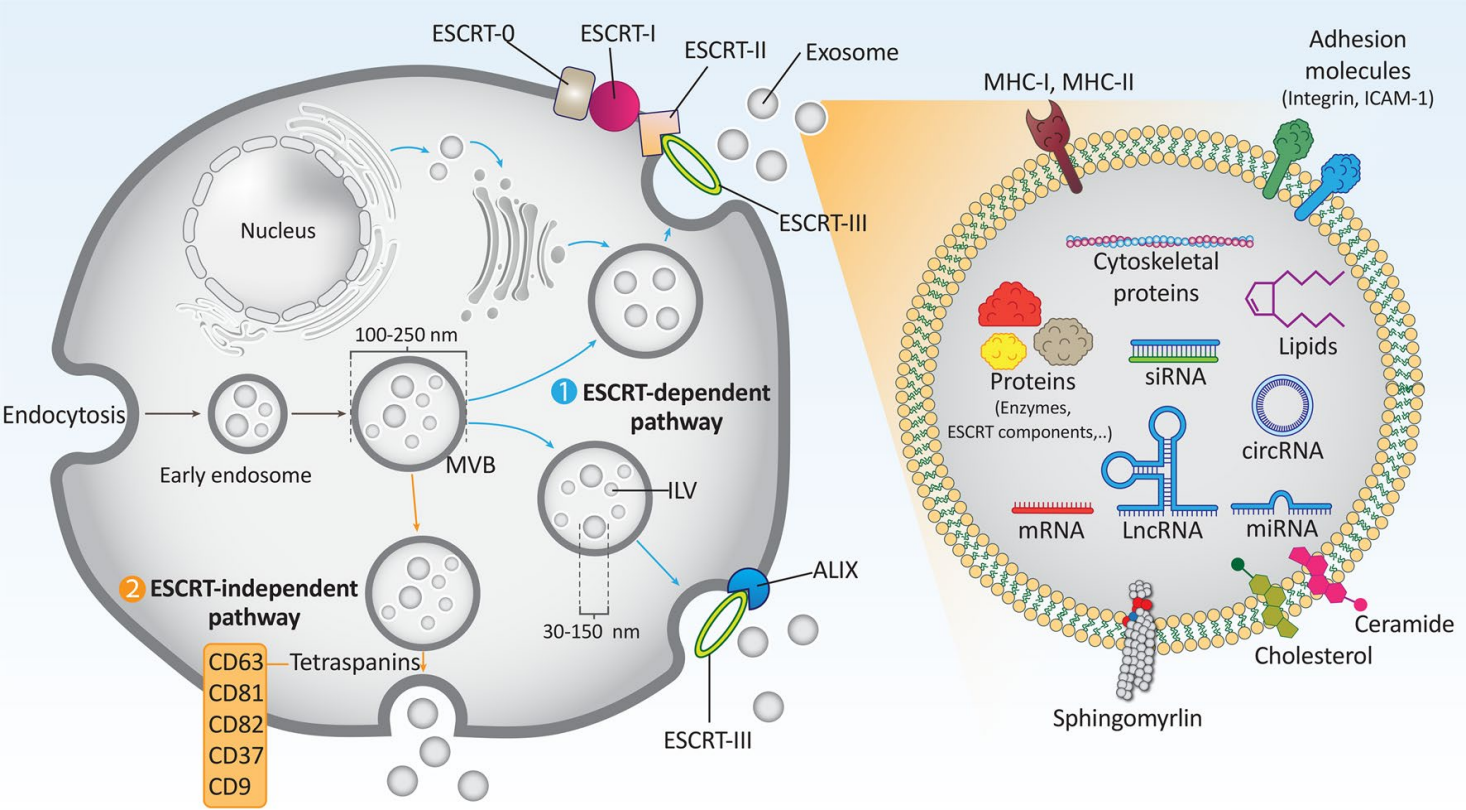
The biogenesis of exosomes. Exosomes contain various types of cargoes such as siRNA, circRNA, lncRNA, mRNA, miRNA, lipids, and proteins, and are therefore involved in various biological mechanisms in cells. They have a particle size of 30–150 nm and various types of proteins shown in the figure may be involved in the biogenesis of exosomes. Targeting these proteins may regulate exosome biogenesis and provide new insights for the development of therapeutics

The preferred mechanism of exosome biogenesis (either ESCRT-dependent or -independent pathway) is determined by cargo and the specific cell type [74]. Two major proteins play notable roles in the transfer and fusion of MVBs: the Ras-associated binding (Rab) family of GTPases and the soluble NSF attachment protein receptor (SNARE) [72, 77, 78]. It is worth noting that some of the MVBs are not fused to cell membranes and are transferred to lysosomes for degradation [72, 77]. The ILVs secreted from MVBs are known as exosomes. There are some limitations to exosome biogenesis and related mechanisms that may be considered in future studies. The underlying mechanism of differentiation of MVBs destined for degradation or fusion with the cell membrane is not known and needs to be studied in detail. Another limitation is the mechanism by which the endocytic system regulates the percentage of MVBs destined for fusion with the cell membrane. In addition, how the sorting of materials from ILVs to MVBs is regulated is still unknown [79]. Figure 1 shows a schematic representation of the biogenesis of exosomes.

## Exosomes and the tumor microenvironment

Most of the tumor mass is occupied by the TME, which comprises the stroma of the tumor [80]. Low oxygen levels, high lactate levels, extracellular acidosis, and poor nutrient content are prominent features of the TME [81, 82]. A variety of cells, including mesenchymal stem cells, fibroblasts, endothelial cells, and immune cells, are present in the TME and can secrete cytokines and growth factors [83]. Cancer-associated fibroblasts are one of the most abundant cells in the TME, creating conditions for tumor growth and progression [84, 85]. The interactions that occur in the TME and the activation/inhibition of signaling networks may determine tumor progression. Therefore, much attention has been devoted to understanding the interactions and developing targeted therapies for the TME [86–88]. This section summarizes the role of exosomes in influencing TME components.

Macrophages are abundant in the TME and have two distinct phenotypes, including M1- and M2-polarized macrophages [89]. Changing the polarization of macrophages toward the M2 phenotype leads to tumor progression and an event that mediates therapy resistance [90, 91]. One of the molecular signaling pathways shown to play an oncogenic role is the signal transducer and activator of transcription 3 (STAT3) pathway [92–96]. A recent experiment attempted to establish a link between STAT3, exosomes, and macrophage polarization in gliomas. The hypoxic state leads to the secretion of exosomes from glioma cells, which subsequently promote cancer progression by inducing M2 polarization of macrophages by triggering autophagy. Exosomes contain high levels of interleukin-6 (IL-6) and miRNA-155-3p. Activation of STAT3 occurs through IL-6, which in turn enhances the expression of miRNA-155-3p to induce autophagy. Due to a positive feedback loop, induced autophagy enhances STAT3 phosphorylation and thus tumorigenesis. Exosome-induced autophagy leads to M2 polarization of macrophages and paves the way for enhanced glioma progression [97]. Similar to glioma, the presence of hypoxia leads to the secretion of exosomes in the TME of colorectal carcinoma. These exosomes contain high levels of miRNA-210-3p, which inhibit apoptosis and promote the transition from G1 to S cycle by downregulating the expression of CELF2. Clinical investigation has also revealed that exosomes containing miRNA-210-3p have high levels in colorectal cancer patients and are correlated with an unfavorable prognosis [98]. Therefore, the signaling networks affected by exosomes may determine tumor progression by influencing TME [99, 100].

Now, the question arises: how can macrophages promote cancer cell migration and invasion? Polarized M2 macrophages are capable of secreting exosomes that promote hepatocellular carcinoma cell metastasis. M2 macrophages-derived exosomes transfer CD11b/CD18 to hepatocellular carcinoma cells. Subsequently, matrix metalloproteinase-9 (MMP-9) is activated, which significantly promotes cancer migration and metastasis [101]. Considering this important role of macrophages in cancer progression, exosomes targeting the TME have been developed. Galectin-9 siRNA was loaded into exosomes and then oxaliplatin was embedded as an antitumor agent and trigger of immunogenic cell death. Exosome-delivered galectin-9 siRNA suppressed M2 polarization of macrophages and oxaliplatin inhibited pancreatic cancer progression [102]. This study demonstrates how exosomes can reprogram the TME in favor of anticancer activity.

**Fig. 2:**
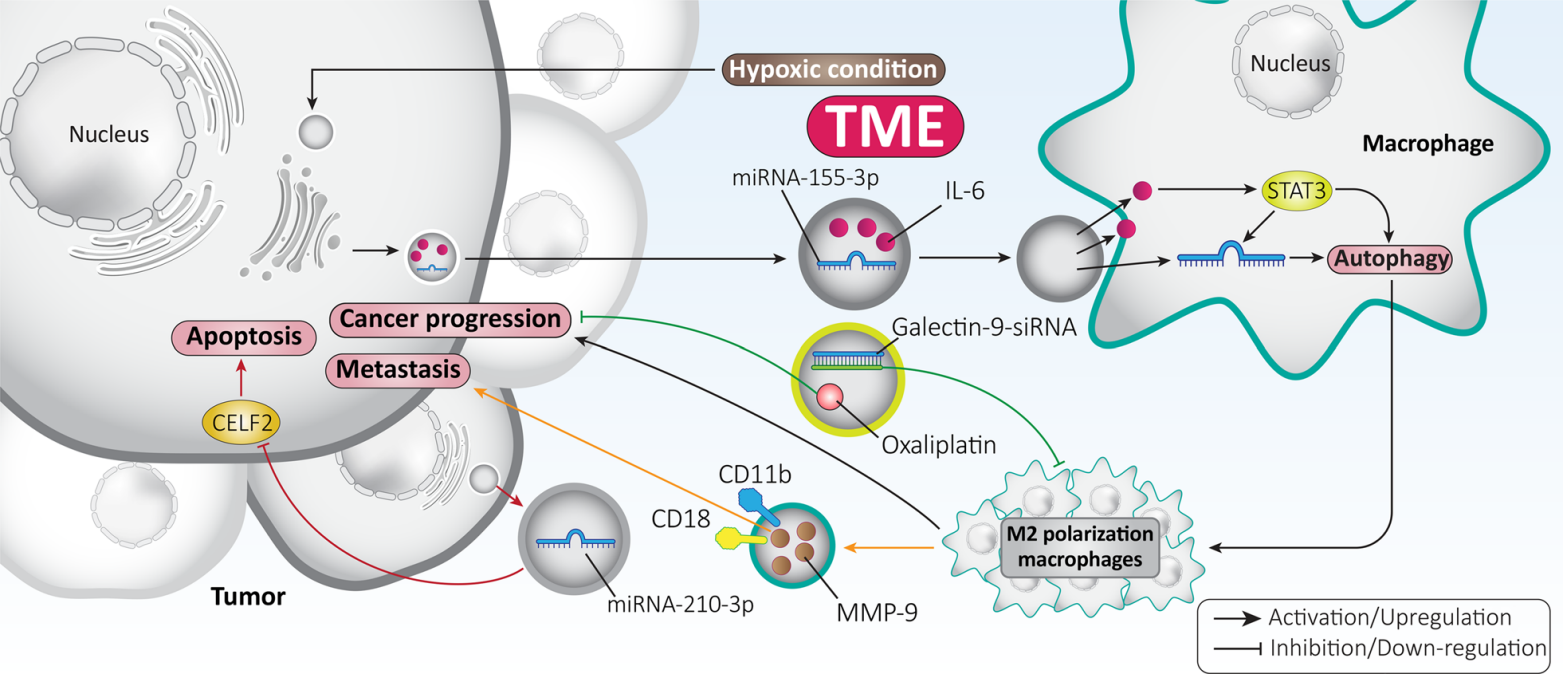
Exosomes in the regulation of the TME. Proliferation and metastasis of tumor cells are strongly modulated by the TME. Exosomes can influence various cellular interactions in the TME and affect tumor progression. In addition, exosomes can transport both anti-tumor agents (oxaliplatin) and siRNA into the TME and modulate tumor growth

Because of the potential of exosomes to affect the TME, efforts have been made to develop exosomes that target the TME and regulate cancer progression. In a recent experiment, exosomes were loaded with manganese carbonyl to mediate their delivery to the TME. This resulted in increased formation of reactive oxygen species (ROS) and was able to reduce tumor proliferation by up to 90% during low-dose radiotherapy [103]. In addition, exosomes that are responsive to inflammatory TME were developed and, because of their ability to cross the blood–brain barrier (BBB), effectively transport doxorubicin into the TME and suppress glioma progression [104]. Therefore, exosomes may be considered promising candidates for targeting the TME and influencing cancer progression. When exosomes are present in the TME, a number of agents such as cytokines can alter their surface. For example, a recent experiment has shown that the surface of exosomes is modified by the CCL2 cytokine via binding to glycosaminoglycan side chains of proteoglycans, altering their cellular uptake and tropism toward certain cells and tissues [105]. Thus, if exosomes are to be manipulated, their interaction with components of the TME and the modification of their cellular uptake should be emphasized. Overall, exosomes exhibit interactions with the TME [106] and further experimentation is needed in basic research, in the development of exosomes for targeting the TME, and also in the introduction of these concepts into clinical courses (Fig. 2).

## Exosomes and tumor angiogenesis

Angiogenesis and vasculogenesis are considered the two most important mechanisms for the formation of new vessels [107–110]. However, there are major differences between angiogenesis and vasculogenesis. Vasculogenesis is involved in the formation of a whole vessel during embryonic development and is responsible for the development of the cardiovascular system. Thus, the endoderm releases vascular endothelial growth factor (VEGF), which induces VEGF receptor 2 (VEGFR2) on mesodermal cells in a paracrine manner [111]. Subsequently, mesodermal cells are converted into angioblasts or endothelial progenitor cells in the periphery of blood islands, whose fusion leads to the formation of primitive capillary networks [112]. Whereas vasculogenesis refers to the formation of new vessels, angiogenesis is the process of vessel formation from preexisting vessels [113]. The process of angiogenesis is inactive in adults and can be observed in physiological processes such as placental angiogenesis and embryo implantation [114–116]. Both angiogenesis and vasculogenesis are critical to the process of wound healing and facilitate this process [117]. Recently, attention has focused on the role of angiogenesis in cancer. Tumor cells should induce angiogenesis to ensure their survival, grow, and spread to different parts of the body. It has been reported that cancer cells cannot grow beyond a size of 1–2 mm if angiogenesis does not occur. Therefore, a promising strategy in cancer treatment could be the inhibition of angiogenesis. The best known factor responsible for the induction of angiogenesis is VEGF, a cytokine involved in cancer progression [118]. The activity of VEGF in neovascularization is related to its binding to receptors such as VEGFR1 and VEGFR2. In addition, VEGF has an affinity for binding to cofactors such as neuropilin-1 (NRP-1) and NRP-2. VEGFR2 expression is mainly observed in endothelial cells, whereas VEGFR1 is found on macrophages, cancer cells, and fibroblasts. The use of monoclonal antibodies is of interest for inhibition of VEGF or VEGFR and suppression of angiogenesis [119].

Since induction of angiogenesis promotes cancer progression, tumor cells secrete exosomes to trigger this mechanism. In this case, multiple molecular signaling pathways are involved that ultimately induce angiogenesis. Oral squamous cell carcinoma (OSCC) cells are able to secrete exosomes containing miRNA-210-3p. Upregulation of miRNA-210-3p occurs in OSCC cells and acts as a tumor-promoting factor by increasing microvessel density (MD) and tumor grade. Mechanistically, exosomal miRNA-210-3p reduces ephrin A3 expression to stimulate the PI3K/Akt axis, trigger angiogenesis, and promote OSCC progression [120]. Indeed, exosomes function as tools of cell–cell communication and can influence the conditions that promote cancer progression. Nasopharyngeal carcinoma (NPC) cells have a high migratory capacity that has been linked to their ability to trigger angiogenesis. Exosomal miRNA-23a binds to the 3’-UTR of TSGA10 and reduces its expression, leading to angiogenesis and increased metastasis of NPC cells [121]. The question now arises: since exosomes are able to regulate angiogenesis, can we isolate exosomes that suppress angiogenesis and thereby impair cancer progression? The answer is affirmative, and such a strategy has already been used in the treatment of lung cancer. It has been reported that exosomes derived from Plasmodium-infected mice inhibit angiogenesis. To test this hypothesis, an animal model of Plasmodium infection was developed in an experiment and then exosomes were isolated for the treatment of lung cancer. These exosomes contained high levels of miRNA-16, -322, -497, and -17, and when injected into a mouse model of lung cancer, there was a significant reduction in the expression of VEGFR2, resulting in inhibition of angiogenesis and reduced tumor progression [122]. This experiment clearly indicates that more studies should be conducted on exosomes and their role in affecting angiogenesis. By developing isolation methods, such exosomes can be obtained and their potential for cancer treatment can be revealed.

In addition to miRNAs, exosomes may also contain lncRNAs involved in the regulation of angiogenesis. In this case, the induction or inhibition of angiogenesis depends on the role of lncRNA as a tumor-suppressor or tumor-promoter. The lncRNA GAS5 is thought to suppress lung cancer progression. Exosomes containing high levels of GAS5 stimulate apoptosis in lung cancer and impair its growth. To this end, exosomal lncRNA GAS5 reduces miRNA-29-3p expression to increase PTEN expression. Subsequently, activated PTEN signaling suppresses PI3K/Akt phosphorylation to inhibit angiogenesis [123]. The role of exosomal ncRNAs in cancer progression will be discussed mechanistically in the next sections. However, it is clear that one way to modulate cancer progression is to influence angiogenesis through exosomes.

Angiopoietin-2 (ANGPT2) is thought to mediate resistance to antiangiogenic therapy by destroying vascular stability and promoting angiogenesis [124]. Suppression of the ANGPT2/Tie2 axis is a promising target [125, 126] because studies have shown the role of this factor in angiogenesis of cancer angiogenesis and in inflammation [127, 128]. Hepatocellular carcinoma (HCC) cells are capable of secreting ANGPT2-containing exosomes. These exosomes are introduced into HUVECs by endocytosis, and increased expression of ANGPT2 induces angiogenesis that promotes cancer progression [129]. As more experiments are performed, the novel signaling networks involved in angiogenesis are revealed. Hypoxia is a common feature of the TME. Recent experiments have shown that hypoxia can induce the secretion of exosomes from tumor cells, which increases their stemness and proliferation rate [130, 131]. A similar phenomenon occurs in colorectal cancer, where hypoxia leads to the secretion of exosomes, which in turn promote both growth and migration of tumor cells. Inhibition of exosome secretion by silencing RAB27a impairs proliferation and growth of colorectal tumors. Under hypoxic conditions, hypoxia-inducible factor-1α (HIF-1α) induces the secretion of exosomes containing Wnt4a. Subsequently, the β-catenin signaling pathway is activated and the nucleus is translocated, leading to angiogenesis and colorectal cancer progression [132].

**Fig. 3:**
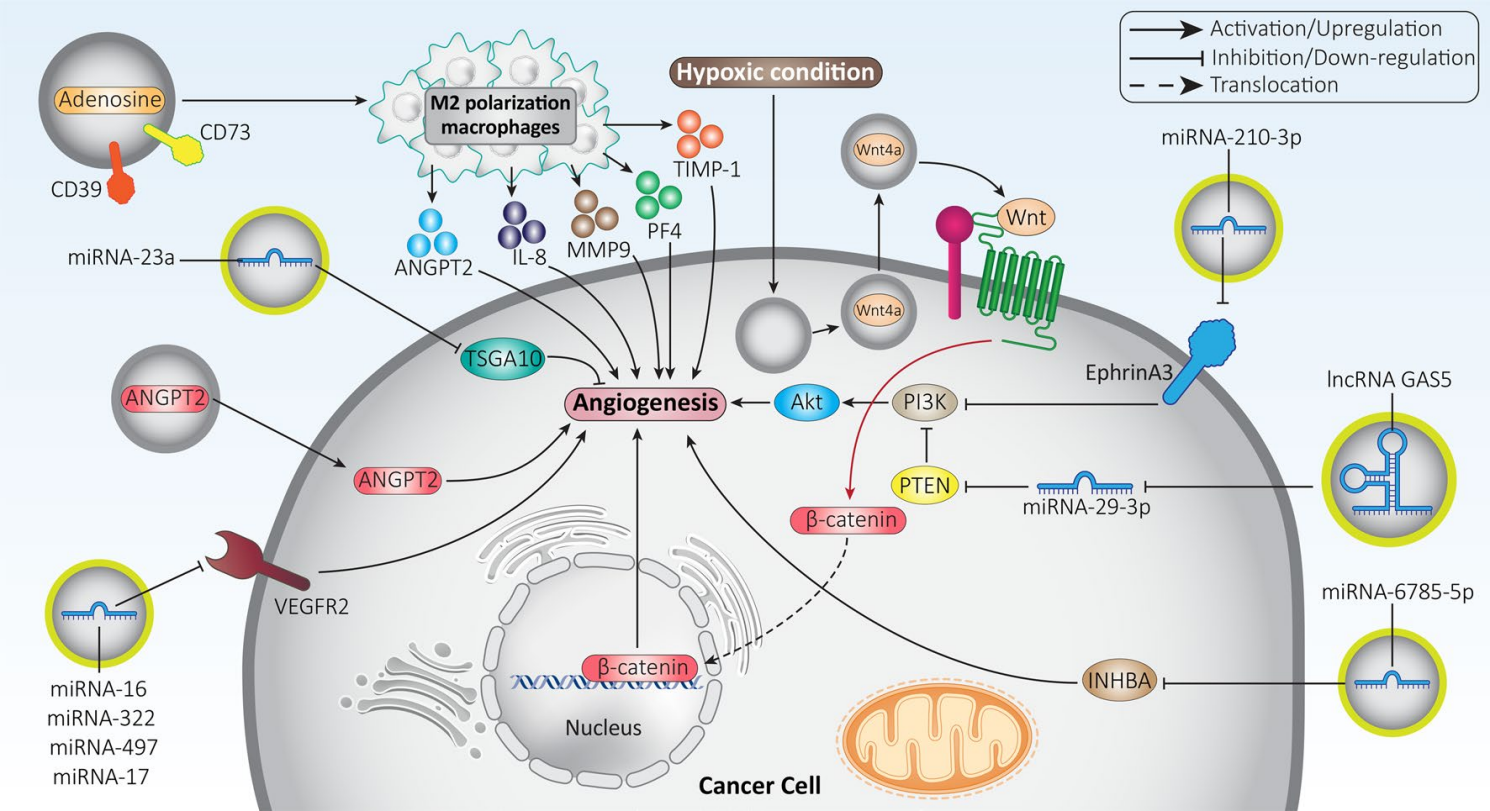
Exosomes in the modulation of angiogenesis in cancer cells. The molecular signaling pathways that regulate angiogenesis, including Akt, PTEN, β-catenin, TSGA10, and ANGPT2, are regulated by exosomes. Induction of angiogenesis promotes tumor progression and therapeutic targeting of exosomes may impair cancer growth

In the previous sections, we have shown that exosomes affect the TME. The interaction of exosomes with the components of the TME may influence angiogenesis and thus cancer progression. Several experiments have shown that macrophages can induce angiogenesis. Recruitment of macrophages can induce angiogenesis to enhance nerve regeneration [133]. In addition, reduction or depletion of macrophages suppresses angiogenesis [134]. Tumor-derived exosomes (TEX) are capable of carrying CD39/CD73 and adenosine, which are enzymatically active. The TEX leads to polarization of macrophages into the M2 phenotype via A_2B_R. Subsequently, M2 macrophages secrete angiogenic factors (ANGPT2, IL-8, MMP9, PF4, and TIMP-1) that induce angiogenesis and promote cancer progression [135]. Overall, the studies are consistent with the fact that angiogenic factors are strongly regulated by exosomes. Depending on the cargo of exosomes, they can act as tumor suppressive or tumor promoting factors to influence angiogenesis in cancer cells. Exosomes can affect various molecular signaling pathways such as MAPK, YAP, VEGF, and miRNAs in modulating angiogenesis in cancer cells (Table 1) [136–141]. Figure 3 illustrates the role of exosomes in regulating angiogenesis in cancer cells.

**Table 1:** Exosomes and their association with angiogenesis in different cancers

Cancer type	In vitro*/*In vivo	Cell line/animal model	Signaling network	Remarks	Refs
Thyroid cancer	In vitro In vivo	Nthy-ori-3–1 cells	miRNA-21-5p/TGFBI miRNA-21-5p/COL4A1	miRNA-21-5p in exosomes is upregulated under hypoxic conditions Angiogenesis is induced TGFBI and COL4A1 are inhibited by miRNA-21-5p to promote angiogenesis and cancer progression	[217]
Esophageal squamous cell carcinoma	In vitro In vivo	ECA109, KYSE410 and HET-1A cell lines Nude mice	–	Angiogenesis is promoted by the increased levels of exosomes under hypoxic conditions	[218]
Head and neck squamous cell carcinoma	In vitro In vivo	PCI-13 (HPV^−^) and UMSCC47 (HPV^+^) cell lines Mouse model	–	Functional reprogramming and phenotypic modulation are observed in endothelial cells Vascular structure formation is increased Proliferation and invasion are promoted Angiogenesis is induced by exosomes carrying angiogenic proteins	[219]
Gastric cancer	In vitro In vivo	SGC7901 cells Xenograft model	miRNA-155/FOXO3a	FOXO3a is inhibited by miRNA-155 in exosomes to induce angiogenesis in gastric to drive cancer progression	[220]
Gastric cancer	In vitro	HUVECs	YB-1/VEGF	Exosomes derived from gastric cancer cells have high levels of YB-1 Apoptosis is inhibited, and metastasis and angiogenesis are enhanced Protein and mRNA levels of VEGF are increased	[221]
Gastric cancer	In vitro	SGC7901 and MGC803 cells	miRNA-6785-5p/INHBA	INHBA expression is reduced by exosomes containing miRNA-6785-5p to impair migration and angiogenesis of cancer cells	[222]
Gastric tumor	In vitro	SGC7901 cells	miRNA-135b/FOXO1	FOXO1 expression is decreased by the overexpression of miRNA-135b in exosomes to induce angiogenesis and exert tumor-promoting effects	[223]
Endometrial cancer	In vitro	SPEC2 and ISK cells	LGALS3BP/PI3K/Akt/VEGFA	Cancer progression is enhanced by exosomes containing LGALS3BP Associated with unfavorable prognosis VEGFA expression is induced by triggering the PI3K/Akt axis Angiogenesis is promoted	[224]
Breast cancer	In vitro	MDA-MB-231, MCF-7 and T47D cells	miRNA-100/mTOR/HIF-1α/VEGF	miRNA-100 is delivered by exosomes in breast cancer therapy Angiogenesis is suppressed to impair cancer progression VEGF expression is downregulated in a time-dependent manner The mTOR/HIF-1α axis is suppressed	[225]
Breast cancer	In vitro	4T1 cells	miRNA-16/VEGF	VEGF is downregulated by exosomes containing miRNA-16 Angiogenesis is suppressed Cancer progression is impaired	[226]
Ovarian cancer	In vitro	A2780 and HO-8910 cells	PKR1/STAT3	Ovarian cancer migration is promoted by exosomes containing PKR1 through induction of angiogenesis Phosphorylation level of STAT3 is increased by PKR1	[227]
Ovarian cancer	In vitro	SKOV3 cells	miRNA-130a	miRNA-130a is delivered by exosomes Angiogenesis is increased to promote cancer progression and trigger drug resistance	[228]
Small-cell lung cancer	In vitro In vivo	H446 cells Xenograft model	Profilin 2	Migration and tube formation capacity of endothelial cells are enhanced Smad2/3 is stimulated by profilin 2 in H446 cells Cancer development and metastasis are enhanced by exosomes	[229]
Colon cancer	In vitro In vivo	HCT-15 cells Nude mice	GDF15/Smad/periostin	Angiogenesis is enhanced by exosomes derived from cancer cells Smad is inhibited by GDF15 to enhance periostin expression to promote angiogenesis	[229]
Colorectal cancer	In vitro In vivo	LoVo and HT29 cells	miRNA-135b-5p	Angiogenesis is triggered, and proliferation and migration are enhanced	[230]
Renal cancer	In vitro	789-0 cells	hepaCAM/VEGF	VEGF is downregulated and angiogenesis is inhibited by exosomes enriched with hepaCAM	[231]
Renal cancer	In vitro In vivo	786-0 cells Nude mice	miRNA-27a/SFRP1/VEGF	SFRP1 is downregulated and VEGF expression is increased by miRNA-27a delivered by exosomes to trigger angiogenesis and promote cancer progression	[232]

## Exosomes and tumor growth

Proliferation of cancer cells increases abnormally and is one of the factors by which cancer cells differ from normal cells. Increased cell cycle progression, inhibition of apoptosis, and glycolysis are considered to be major main factors in cancer growth [142–146]. The aim of this section is to evaluate the role of exosomes in modulating proliferation of cancer cells. Rapidly dividing cancer cells require high levels of energy to sustain their proliferation. Oxidative phosphorylation is a slow process and cannot provide tumor cells with energy to meet their needs. Therefore, a switch in metabolism from oxidative phosphorylation to glycolysis is initiated. Therefore, suppression of glycolysis can be considered a promising strategy in cancer therapy [147–150]. Exosomes have been shown to increase the growth rate of lung cancer cells via the induction of glycolysis. To this end, exosomes were isolated from irradiated lung cancer cells. They exhibited high levels of ALDOA and ALDH3A1, which stimulate glycolysis to increase lung cancer proliferation [151]. With regard to the close link between drug resistance and glycolysis, studies have attempted to elucidate a link between exosomes, glycolysis, and chemoresistance. Macrophages are capable of secreting exosomes that play a tumor-promoting role. A recent experiment has shown that macrophage-derived exosomes enhance glycolysis, mediating lung cancer cell resistance to cisplatin chemotherapy. Molecular pathway study shows that these exosomes have high levels of miRNA-3679-5p and decrease the expression of NEDD4L to promote the stability of c-Myc, leading to lung cancer growth, induction of glycolysis, and mediation of cisplatin resistance [151]. Therefore, exosomes can induce glycolysis in favor of cancer growth and induce drug resistance [152].

STAT3 signaling is an oncogenic pathway that inhibits apoptosis and cell cycle arrest and promotes growth and metastasis [153]. STAT3 induces EMT and promotes cancer invasion. Overexpression of STAT3 is associated with poor prognosis and triggers chemoresistance [154–158]. Exosomes alter the polarization of macrophages and transform them into cancer-associated macrophages. The exosomes are enriched in gp130 and induce STAT3 signaling via IL-6 upregulation [159]. STAT3-containing exosomes are able to promote ovarian cancer progression by inducing an imbalance between T cells and tumor-associated macrophages in favor of immunosuppression [160]. Cyclin D1, MMP-2, and MMP-9 are upregulated by STAT3-containing exosomes and promote proliferation and invasion of breast cancer cells [161]. Similarly, hypoxic conditions in the TME enhance the ability of colon cancer cells to self-proliferate by upregulating STAT3 expression [162]. Overall, several molecular signaling pathways are affected by exosomes, and understanding their interaction may pave the way for the development of novel therapeutics [163–165].

Apoptosis is an important signaling pathway regulated by exosomes in tumors. Inhibition of apoptosis may pave the way for tumor progression and resistance to therapy [166]. A recent experiment has shown that cancer-associated fibroblasts secrete exosomes containing miRNA-92a-3p, which act as a tumor-promoting factor and induce the Wnt/β-catenin axis, leading to inhibition of mitochondrial apoptosis and inducing resistance of colorectal cancer cells to 5-fluorouracil [167]. The ROS can induce apoptosis in cancer cells. It has been reported that modulation of the levels of ROS may be important for the response of cancer cells to therapy [168–171]. In pancreatic cancer, exosomes containing miRNA-155 reduce the expression of DCK, an enzyme involved in the metabolism of gemcitabine. This is followed by an increase in superoxide dismutase and catalase, leading to a reduction in ROS and subsequent growth of cancer cells and mediating their resistance to chemotherapy [172, 173]. Interestingly, not only can apoptosis in cancer cells reduce their proliferation, but apoptosis in immune cells can also affect cancer progression. A recent experiment has shown that pancreatic cancer-derived exosomes are taken up by lymphocytes to induce p38 MAPK signaling and mediate apoptosis triggered by endoplasmic reticulum stress apoptosis to stimulate immunosuppression and pave the way for cancer progression [174]. On the other hand, exosomes derived from colorectal cancer cells stimulate extracellular signal-regulated kinase (ERK) to suppress apoptosis and promote growth [175]. Apoptosis as a mechanism of programmed cell death is closely related to autophagy. In general, autophagy is involved in cell homeostasis by degrading aged and toxic organelles and macromolecules. However, the activation of autophagy in cancer cells is controversial and requires further clarification because it plays both tumor-promoting and tumor-suppressive roles [176–179]. A recent experiment has shown that sirtuin 2 (SIRT2) increases the mRNA stability of transcription factor EB (TFEB) and induces the release of exosomes to trigger autophagy and decrease apoptosis in non-small cell lung cancer cells [180]. Therefore, special attention should be paid to autophagy in cancer progression when studying apoptosis regulation by exosomes. Overall, studies support the fact that exosomes can either increase or decrease cancer cell proliferation [181–185].

## Exosomes and tumor metastasis

Cancer cell invasion threatens the lives of many cancer patients around the world by enabling the spread of tumor cells to various organs and tissues of the body and mediating their malignancy [186–188]. Therefore, the factors involved in cancer metastasis should be highlighted to direct future experiments to target them [189–192]. Exosomes have been shown to be critical regulators of cancer metastasis. RelA and RelB are able to decrease the levels of MCAM and CD146 adhesion molecules in the release of EVs, leading to breast cancer metastasis. Silencing of RelA and RelB decreases the organotropic ability of exosomes in vivo and significantly reduces their ability to promote breast cancer migration and invasion [193]. It appears that exosomes containing Eph receptor A2 (EphA2) are able to transfer metastatic potential to pancreatic cancer cells and promote their invasion [194]. In contrast, there are exosomes capable of suppressing the cancer cell metastasis. For example, migration and invasion of non-small cell lung cancer cells were significantly decreased by miRNA-let7e-containing exosomes. LSD1 is upregulated in lung cancer and reduces E-cadherin levels to promote migration. Exosomes containing miRNA-let7e are able to increase CDH1 expression via LSD1 down-regulation to impair lung cancer metastasis [195]. Therefore, exosomes are important modulators of cancer migration and invasion.

The molecular mechanisms responsible for cancer migration and invasion are influenced by exosomes. The epithelial-to-mesenchymal transition (EMT) is among the best known mechanisms involved in cancer migration and invasion [196]. The decrease in E-cadherin, and the increase in N-cadherin and vimentin mediate EMT-induced metastasis in cancer cells [197, 198]. There are a number of factors known as EMT-inducing transcription factors (EMT-TFs), including ZEB1/2, TGF-β, Snail, Slug, and Twist, which can stimulate EMT in cancer cells and promote tumor invasion [199, 200]. A recent experiment has shown that exosomes containing the integrin alpha 2 subunit (ITAG2) are able to induce EMT and enhance prostate cancer cell metastasis [201]. On the other hand, exosomes containing miRNA-204 exhibit anti-tumor activity and reduce lung tumor cell invasion and migration by inhibiting EMT. To this end, exosomal miRNA-204 reduces the expression of KLF7 to inhibit the Akt/HIF-1α axis, resulting in a reduction of lung cancer migration and invasion by inhibiting EMT [202]. The ascites of ovarian cancer secretes exosomes containing miRNA-6780b-5p, which increase cancer migration and invasion in patients. It appears that exosomes containing miRNA-6780b-5p induce EMT to promote ovarian cancer metastasis [203]. The ability of exosomes to inhibit or induce EMT depends on their cargo. For example, miRNA-381-3p plays a tumor-suppressive role and exosomes containing this miRNA suppress EMT-mediated metastasis of breast cancer cells [204]. Therefore, there is increasing evidence for the role of exosomes in regulating metastasis by targeting the EMT mechanism [205, 206].

**Fig. 4:**
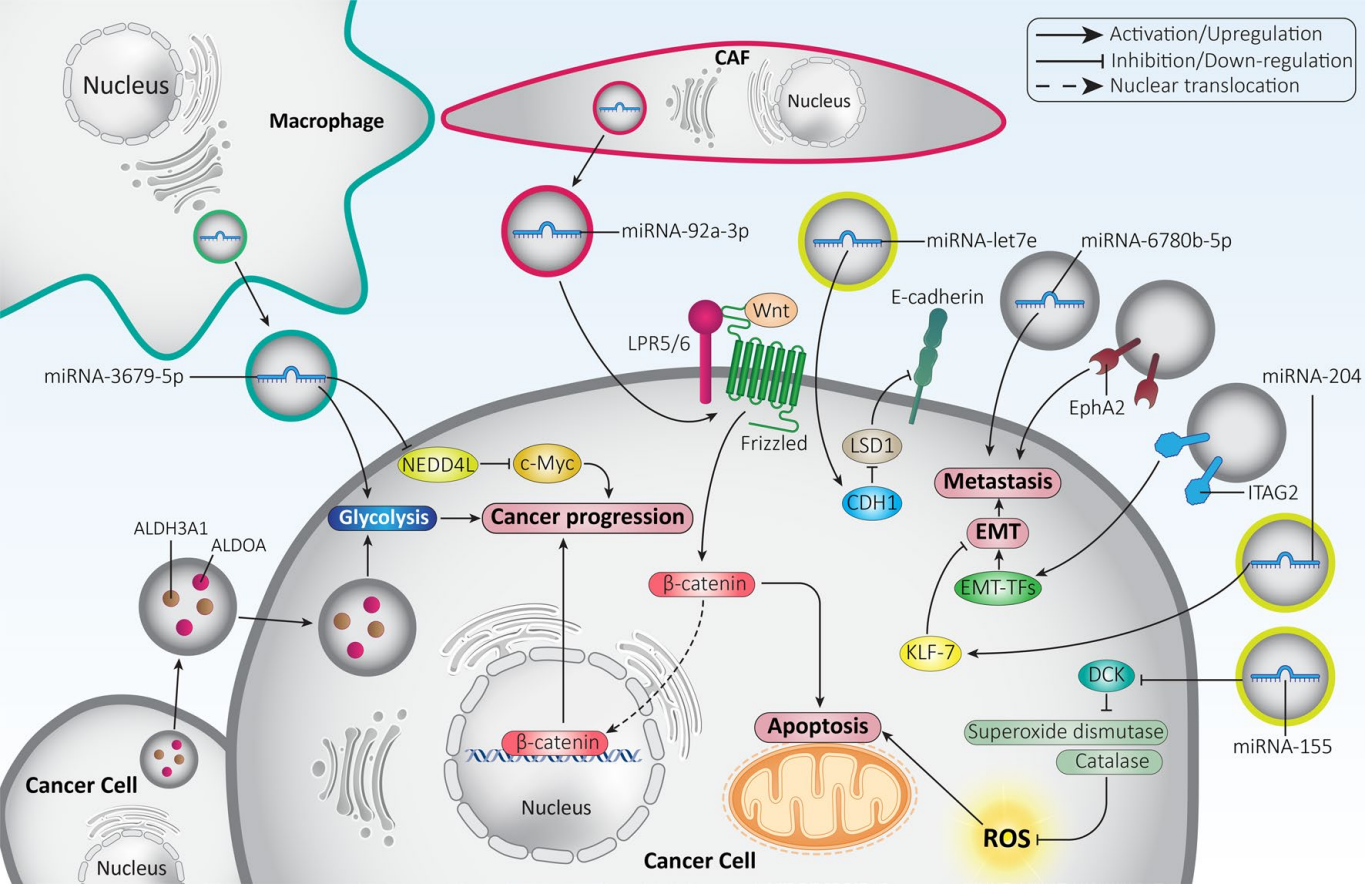
Exosomes in the regulation of cancer cell growth and invasion. Glycolysis responsible for tumor growth is regulated by exosomes. CAFs are able to secrete exosomes to modulate tumor progression. EMT, metastasis, ROS and apoptosis are other signaling pathways affected by exosomes in tumor cells

In addition to EMT, matrix metalloproteinases (MMPs) are also involved in increasing cancer metastasis [207–211], and recent experiments have confirmed this. MMP-2 induces EMT to increase squamous cell carcinoma metastasis, and it may act as an independent factor in patient prognosis [212]. A clinical experiment demonstrated overexpression of MMP-7 in bladder cancer, which is associated with unfavorable prognosis and shortened overall survival of patients [213]. Moreover, MMP-3 is involved in the induction of angiogenesis, which promotes cancer progression [214]. Therefore, suppression of MMP activity may be of interest to inhibit cancer metastasis. Overexpression of trefoil factor 3 (TFF3) leads to upregulation of MMP-2 and MMP-9, enhancing prostate cancer cell invasion. Mesenchymal stroma cell-derived exosomes containing miRNA-143 exhibit anti-tumor activity and inhibit TFF3 to downregulate MMP-2 and MMP-9, leading to suppression of metastasis [215]. In contrast, exosomes derived from renal cancer cells are able to increase the expression of MMP-9 to promote invasion [216]. Although some studies have focused on the interaction between exosomes and MMP, there is still a long way to go to uncover the signaling networks involved (Fig. 4).

## Exosomes and cancer resistance

### Drug resistance

In the field of cancer therapy, a variety of antitumor agents have been developed, including cisplatin, 5-fluorouracil (5-FU), sorafenib, and oxaliplatin [233]. However, long-term use of these chemotherapeutic agents leads to drug resistance and an unfavorable prognosis for cancer patients. A specific mechanism is responsible for chemoresistance. Among others, drug efflux, upregulation of anti-apoptotic factors, DNA damage repair, epigenetic changes, and the TME may influence drug resistance [234–239]. The current section focuses on the potential role of exosomes in drug resistance of cancer cells.

A growing body of evidence suggests that exosomes are capable of influencing the response of cancer cells to chemotherapy [240]. The ability of exosomes to transport cargoes has made them promising agents in cancer chemotherapy. As nanostructures, exosomes can mediate the co-delivery of a miRNA-21 inhibitor and 5-FU in colon tumor chemotherapy. The 5-FU and miRNA-21 inhibitor were loaded into exosomes via electroporation. Systematic administration of exosomes containing the miRNA-21 inhibitor and 5-FU suppressed tumor growth in mice. Exosomes administration enhances cellular uptake and reduces miRNA-21 expression in favor of colon cancer suppression. Moreover, miRNA-21 inhibitor and exosomes loaded with 5-FU induce cell cycle arrest and apoptosis. These anti-tumor activities are mediated via the upregulation of PTEN and hMSH2 as tumor suppressor factors in colon cancer [241]. The process of exosome secretion, cargo transport, and involvement in drug resistance are complex and should be elucidated. The epithelial ovarian cancer cells are able to recruit macrophages and stimulate their tumor-associated phenotype. Hypoxia in the TME leads to the secretion of exosomes from macrophages containing high levels of miRNA-223 as a tumor-promoting factor. The process of mediating drug resistance is that miRNA-223 delivered by exosomes reduces PTEN expression to induce PI3K/Akt signaling. To establish a link between hypoxia and exosome secretion, patients with ovarian cancer were studied. It was found that overexpression of HIF-1α, a hypoxia marker, occurs in ovarian cancer patients and is associated with upregulation of miRNA-223. Therefore, complicated molecular pathways and mechanisms are involved in the secretion of exosomes and the triggering of chemoresistance [242]. Another experiment demonstrates the potential role of macrophage-derived exosomes in triggering drug resistance in pancreatic cancer. An interesting point is that exosomes may be involved in the inactivation of chemotherapeutic agents in triggering drug resistance. Macrophage-derived exosomes contain miRNA-365 as a tumor-promoting factor and are able to induce gemcitabine resistance in pancreatic cancer. To this end, exosome-derived miRNA-365 stimulates the cytidine deaminase enzyme to inactivate gemcitabine, leading to chemoresistance in pancreatic cancer [243].

In addition to inactivating chemotherapeutic agents, exosomes can direct cancer cells toward cell death. It has been reported that exosomes can be obtained from CSCs in pancreatic cancer. These exosomes contain miRNA-210, which can induce gemcitabine resistance via inducing mTOR signaling. Moreover, these exosomes suppress gemcitabine-mediated apoptosis and cell cycle arrest [244]. Consequently, various signaling networks are affected by exosomes in triggering chemoresistance. In addition, the accumulation of chemotherapeutic agents in tumor cells is impaired. Exosomes are able to induce efflux of cisplatin from ovarian cancer cells under hypoxic conditions, demonstrating that they can prevent internalization of chemotherapeutic agents. Furthermore, STAT3 plays an important role in this case. Overexpression of STAT3 in hypoxic condition is crucial for exosome release and triggering cisplatin resistance in ovarian cancer. Suppression of STAT3 signaling alters the levels of Rab7 and Rab27a proteins, preventing the secretion of exosomes [245].

**Fig. 5:**
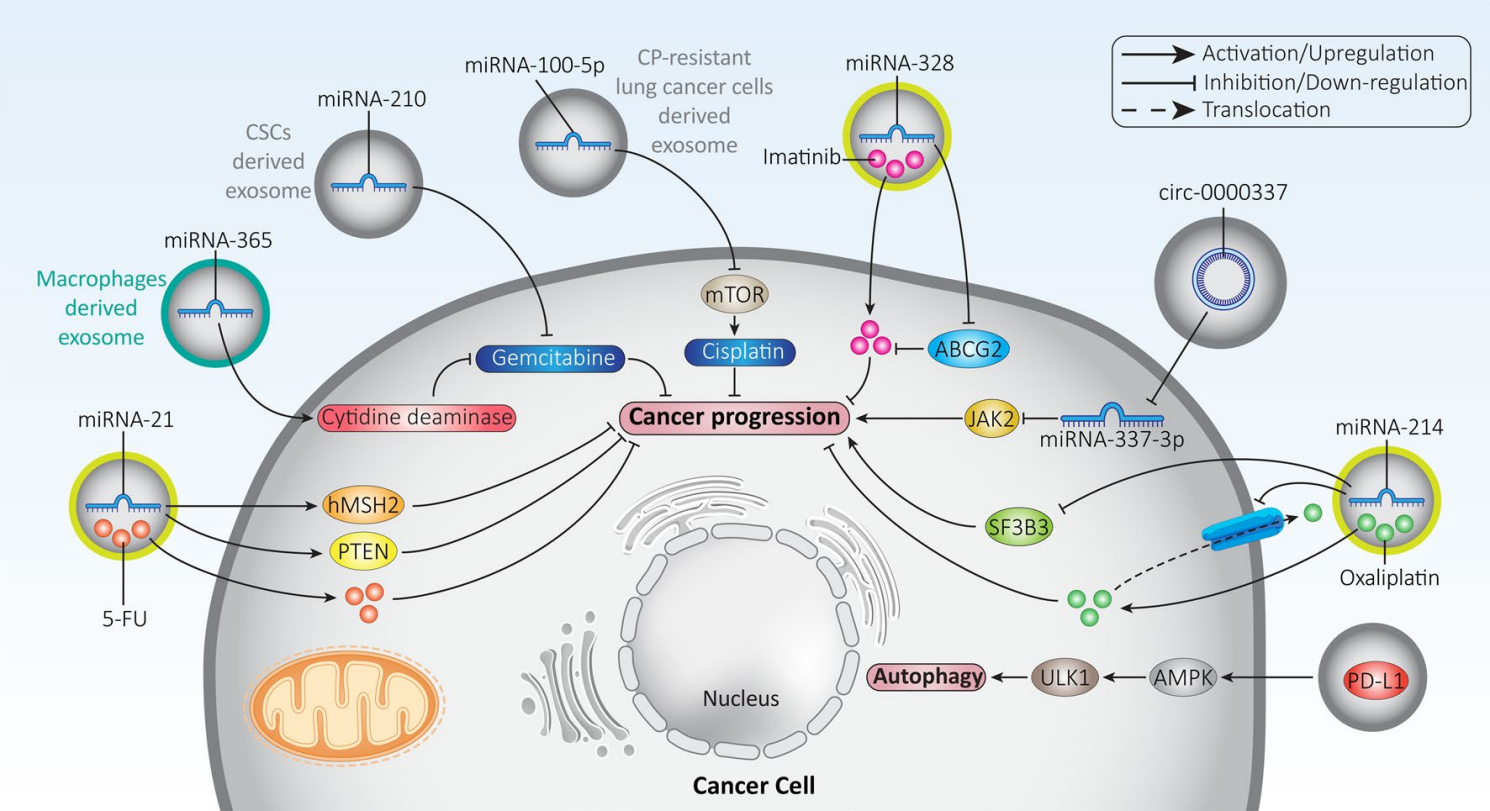
The role of exosomes in modulating the response to drug therapy. Most experiments focused on exosomal miRNAs and their downstream targets such as PTEN and JAK2. PTEN suppresses cancer progression, while JAK2 promotes cancer malignancy. Depending on the function of each molecular mechanism, the role of exosomes in cancer progression or inhibition varies

Tumor cells exhibiting features of drug resistance are able to secrete exosomes that accelerates chemoresistance. Such a strategy has been studied in lung cancer. Exosomes derived from cisplatin-resistant lung cancer cells have high levels of miRNA-100-5p, which decrease the expression of mTOR, leading to cisplatin resistance [246]. In addition, exosomes may act as a means of communication between normal and cancer cells in inducing drug resistance. Endothelial cells are able to secrete exosomes with a particle size of 40–100 nm, which trigger EMT-mediated metastasis in nasopharyngeal carcinomas and mediate their resistance to chemotherapy [247]. Exosomes can be used to suppress chemoresistance. In one experiment, exosomes were used to deliver anti-miRNA-214 to gastric cancer cells to induce apoptosis and decrease proliferation and invasion, leading to drug sensitivity [248]. Overall, the studies are consistent with the fact that exosomes can affect the growth and invasion of cancer cells to influence their response to chemotherapy. They contain various cargoes and can modulate molecular signaling pathways in favor of chemoresistance or chemosensitivity. Such exosomes and associated signaling networks should be elucidated to prevent chemoresistance in cancer cells [249–259]. Table 2 provides an overview of exosomes and their association with drug resistance in cancer. Figure 5 shows a schematic representation of exosomes in regulating drug action.

**Table 2:** Exosomes and their function in mediating drug resistance/sensitivity in cancer

Cancer type	Chemotherapeutic agent	Signaling network	Remark	Refs
Breast cancer	Adriamycin	–	Drug resistance is induced by the transfer of P-gp and UCH-L1 proteins through exosomes into the extracellular microenvironment	[260]
Breast cancer	Anthracycline and taxane agents	–	Chemoresistance is observed in breast cancer patients who had high levels of GSTP1-containing exosomes	[261]
Breast cancer	Adriamycin	MDR1 P-glycoprotein	Drug resistance is induced by exosomes by enhancing the expression of MDR1 and P-glycoprotein Chemoresistance is inhibited by suppression of exosome formation and secretion by psoralen	[262]
Breast cancer	Gemcitabine	Autophagy EMT/HIF-α	Autophagy is inhibited by exosomes containing siMTA1 EMT is suppressed Tumor growth in vitro and in vivo is retarded	[263]
Liver cancer	Sorafenib	–	Selectivity of exosomes against cancer cells is increased by modifying the surface of exosomes Drug resistance is suppressed by synergistic cancer chemotherapy with sgIQ 1.1 plasmid-loaded exosomes	[263]
Leukemia	Etoposide	Bax Bcl-2 PARP Caspase-3	Drug resistance is induced by exosomes derived from bone mesenchymal stem cells by increasing the expression of Bcl-2 and decreasing the expression of Bax, caspase-3, and PARP	[264]
Leukemia	Imatinib	Bax Bcl-2 Caspase-3 Caspase-9	Apoptosis is prevented by exosomes derived from mesenchymal stromal cells, and leukemia cell survival is increased The expression of Bax, caspase-3 and caspase-9 is downregulated, and the expression of Bcl-2 is increased	[265]
Leukemia	Imatinib	miRNA-328/ABCG2	Drug sensitivity is increased by decreasing ABCG2 expression through miRNA-328 in exosomes	[266]
Glioblastoma	Temozolomide	PD-L1/AMPK/ULK1/autophagy	Autophagy is induced by the exosomes containing PD-L1 through stimulation of the AMPK/ULK1 axis, which mediates drug resistance	[267]
Glioblastoma	Temozolomide	STAT3/miRNA-21/PDCD4	STAT3 is downregulated by a combination of temozolomide and pacritinib miRNA-21 expression is reduced to upregulate the PDCD4 tumor suppressor M2 polarization of macrophages is inhibited Glioblastoma tumorigenesis is prevented	[268]
Non-small cell lung cancer	Cisplatin	miRNA-146a-5p	Low levels of miRNA-146a-5p are observed in cisplatin-resistant A549 cells and can be used to predict cancer recurrence	[269]
Oral cancer	Cisplatin	miRNA-155/FOXO3a	FOXO3a expression is enhanced by exosomes containing the miRNA-155-inhibitor Mesenchymal-to-epithelial transition is triggered to suppress cancer cell migration and invasion	[270]
Hepatocellular carcinoma	Oxaliplatin	miRNA-214/P-gp miRNA-214/SF3B3	P-gp and SF3B3 expression is decreased by exosomal miRNA-214 Drug sensitivity is increased	[271]
Hepatocellular carcinoma	Cisplatin	miRNA-199a-3p	Drug resistance is suppressed by apoptosis induction through the increased expression of miRNA-199a-3p delivered by exosomes	[272]
Prostate cancer	Docetaxel	CD44v8-10 mRNA	Drug resistance is mediated by the presence of CD44v8-10-containing exosomes in the serum of prostate cancer patients	[273]

### Radio-resistance

Radiotherapy is another cancer treatment option that uses radiation to inhibit cancer progression and induce cell death [274]. However, due to specific conditions in the TME such as hypoxia, cancer cells could develop resistance to radiotherapy, and the factors involved in this phenomenon should be elucidated [275, 276].

Most experiments have focused on the relationship between exosomes and drug resistance. However, there are also a few studies examining the role of exosomes in radioresistance. For example, a recent experiment has shown that cancer-associated fibroblasts are able to secrete exosomes to promote stemness of colorectal tumors and trigger their clonogenicity and radioresistance. Mechanistically, these exosomes induce transforming growth factor-beta (TGF-β) to mediate radioresistance. When this signaling pathway is suppressed using antibodies, colorectal tumor progression is impaired and sensitivity to radiotherapy is increased [277]. In contrast, there are exosomes capable of suppressing radioresistance. Exosomes containing miRNA-34c suppress proliferation, invasion, and EMT in nasopharyngeal carcinomas. In addition, miRNA-34c-loaded exosomes induce apoptosis and mediate radiosensitivity. Molecular pathway study shows that miRNA-34c-loaded exosomes suppress the β-catenin signaling pathway, thereby increasing the sensitivity of nasopharyngeal carcinoma cells to radiotherapy [278]. In the previous section, it was shown that chemotherapy of cancer cells induces the secretion of exosomes. Moreover, chemoresistant cancer cells are capable of secreting exosomes, which favors their progression and promotes drug resistance [279, 280]. A recent experiment has shown that exosomes can be obtained from irradiated gastric cancer cells [281]. However, further studies are needed to determine whether exosomes are involved in the development of radioresistance.

### Immune evasion and inflammation

Although few experiments have investigated the role of exosomes in immune resistance and evasion, these studies show that exosomes are promising candidates in this case because of their modulatory effect on immune cells. The T-regulatory cells (Treg cells) are well known because of their immunosuppressive effects. In breast cancer, the CD73 + Treg cells are able to facilitate immune evasion by producing adenosine. The exosomes containing the lncRNA SNHG16 increase the expression level of CD73 on Treg cells. To this end, exosomal SNHG16 decreases the expression of miRNA-16-5p via sponging to induce the TGF-β/SMAD5 axis, resulting in overexpression of CD73 on Treg cells. Therefore, the ability of exosomes to transmit SNHG16 may mediate overexpression of CD73 on Treg cells and lead to immunosuppression in breast cancer [282]. Programmed death-1 (PD-1) is a molecular pathway that causes T cell exhaustion and prevents their proliferation. Moreover, PD-1 induces apoptosis in cytotoxic T cells and inhibits their anti-tumor activity to mediate immune evasion. Binding of PD-L1 to PD-1 triggers this pathway [283]. A recent experiment has shown that exosomes derived from cancer-associated fibroblasts contain high levels of miRNA-92 as a tumor-promoting factor. Exosomal miRNA-92 mediates the interaction between LATS2 and YAP1 in breast cancer cells. Subsequently, YAP1 translocates to the nucleus and binds to the PD-L1 promoter to enhance its expression, leading to the apoptosis of T cells and a decrease in proliferation of these cytotoxic cells [284]. Exosomes can not only evade immune defences but also influence immune cells to promote cancer progression. Indeed, interactions between exosomes and immune cells can create optimal conditions for increased cancer growth and invasion. NF-κB signaling is related to the inflammatory process and may promote cancer progression. NF-κB expression in cancer is regulated by other molecular signaling pathways, of which miRNAs are the best known [285]. On the other hand, there is growing evidence that chronic inflammation and pro-inflammatory cytokines promote cancer progression [286–288]. A recent experiment has shown that exosomes derived from breast cancer cells have high levels of miRNA-183-5p and are able to decrease the expression of PPP2CA. Decreased expression of PPP2CA paves the way for triggering NF-κB signaling and mediating chronic inflammation. In addition, this signaling network increases the levels of pro-inflammatory cytokines such as IL-1β, IL-6 and TNF-α. Therefore, the transmission of miRNA-183-5p by exosomes and its effect on inflammation may promote the proliferation and invasion of breast cancer cells [289].

TGF-β mediates immune evasion of breast cancer cells. To this end, TGF-β increases the levels of PD-L1 in exosomes and stimulates the dysfunction of cytotoxic CD8 T cells [290]. Another experiment shows that exosomes derived from multiple myeloma suppress apoptosis and increase the growth rate of Treg cells, triggering immune dysfunction [291]. In addition, exosomes are able to promote the progression of gastric cancer by suppressing the maturation of dendritic cells [292]. Exosomes containing indoleamine 2,3-dioxygenase (IDO) may induce T-cell dysfunction via triggering the IL-6/TNF-α axis [293]. Future experiments may focus on targeting exosomes in preventing immune evasion and suppressing inflammation to impair cancer progression [294, 295].

## Exosomal miRNAs

miRNAs are endogenous, short noncoding RNAs with a length of 19–24 nucleotides that can regulate gene expression at the posttranscriptional level by binding to the 3’-untranslated region (3’-UTR) of target mRNAs [296, 297]. Recent experiments have shed light on the role of miRNAs in cancer. For example, hypoxic conditions enhance lung cancer progression by decreasing the expression of miRNA-495 and miRNA-5688 and subsequently increasing IL-11 levels [298]. Moreover, decreased expression of miRNA-100 and miRNA-125b paves the way for overexpression of IGF2 and subsequent cancer stem cell features in hepatocellular carcinoma [299]. Further studies have shown that miRNAs can be considered as reliable biomarkers for cancer diagnosis [300, 301]. Because exosomes are capable of transmitting miRNAs, we dedicated this section to the study of exosomal miRNAs in the regulation of cancer progression.

In the previous sections, the role of exosomes in cancer progression has been clearly demonstrated as they affect the TME and the therapeutic response of cancer cells. It has been discussed that exosomes may contain various genes that influence cancer progression. The current section focuses specifically on exosomal miRNAs and how they may modulate cancer progression. In a recent experiment, exosomes were isolated exosomes by centrifugation from colorectal cancer cells infected with *Fusobacterium nucleatum* and transferred to uninfected cancer cells. The exosomes were found to contain high levels of miRNA-1264, miRNA-92b-3p, and miRNA-27a-3p, which are able to enhance metastasis and tumor stage of colorectal cancer [302]. There is increasing evidence that hypoxic conditions in the TME significantly promote carcinogenesis in gastric cancer [303, 304]. It appears that hypoxia induces the secretion of exosomes from gastric cancer cells. These exosomes promote both growth and migration of gastric cancer. These exosomes contain miRNA-301a-3p, which acts as a tumor-promoting factor and increases the stability of HIF-1α and inhibits its degradation by targeting PDH3 and hydroxylating HIF-1α subunits. Moreover, there is a positive feedback loop between HIF-1α and miRNA-301a-3p in enhancing the proliferation and invasion of gastric cancer cells. Clinical investigation shows that exosomal miRNA-301a-3p is upregulated in gastric cancer patients and mediates peritoneal metastasis [305].

The PI3K/Akt axis is a trigger of cancer progression and its induction promotes cancer cell proliferation and invasion [306–308]. In addition, the PI3K/Akt axis enhances the aggressive behavior of cancer and is associated with drug resistance. PTEN is the negative regulator of the PI3K/Akt axis and increasing its expression is a promising strategy to interrupt cancer progression [309–311]. A recent experiment shows that exosomes containing miRNA-22-3p have a tumor suppressive effect and prevent colorectal cancer progression by downregulating PI3K/Akt [312]. In addition to increased proliferation, exosomes may facilitate the transfer of apolipoprotein E between cells to induce the PI3K/Akt axis, which mediates cytoskeletal remodeling and promotes gastric cancer cell migration and invasion [313]. Therefore, the PI3K/Akt axis is strongly regulated by exosomes [314]. The question now arises: is there a link between exosomes and PTEN as upstream mediators of the PI3K/Akt axis? The answer is positive, and this potential has been confirmed in several experiments. In non-small cell lung cancer, exosomes containing miRNA-126 are able to enhance PTEN expression in suppressing the PI3K/Akt axis and impair metastasis. The in vivo experiment showed that miRNA-126 reduced lung cancer metastasis by modulating the PTEN/PI3K/Akt axis [315]. On the other hand, hypoxia leads to the secretion of exosomes from colorectal cancer cells. The exosomes contain high levels of miRNA-410-3p, which decrease PTEN expression to induce the PI3K/Akt axis and promote cancer cell invasion. Moreover, exosomal miRNA-410-3p is associated with unfavorable prognosis of colorectal cancer patients [316]. The growth rate of esophageal cancer cells is significantly increased by the transfer of miRNA-93-5p through exosomes and the downregulation of PTEN [317].

Another molecular signaling pathway involved in cancer progression is the Wnt/β-catenin axis. Nuclear translocation of β-catenin promotes cancer growth and invasion [318–320]. Activation of Wnt signaling is associated with poor prognosis. In addition, the Wnt/β-catenin axis can mediate features of drug resistance in cancer cells [321–325]. Exosomes are able to induce Wnt5b signaling, thereby increasing the progression of lung cancer cells [326]. The exosomes containing miRNA-320a act as tumor-promoting factors and reduce the expression of SOX4. As a result, Wnt/β-catenin activation occurs, which significantly promotes the growth and metastasis of lung cancer cells [327]. Another experiment shows that exosomes from breast cancer cells contain high levels of miRNA-146a, which reduces the expression of TXNIP to induce the Wnt/β-catenin axis, leading to activation of cancer-associated fibroblasts in the TME and promoting breast cancer progression [328]. Therefore, regulation of Wnt signaling by exosomes modulates cancer progression [329].

One of the increasing challenges in breast cancer is bone metastasis, which is associated with pain, decreased overall patient survival, and an unfavorable prognosis. Therefore, efforts have been made to uncover the role of exosomal miRNAs in bone metastasis of breast cancer cells in order to target them in future experiments. A clinical study has shown that serum exosomes containing miRNA-21 promote bone metastasis in breast cancer patients [330]. Although these studies demonstrate the tumor-promoting role of exosomal miRNAs, there are also experiments showing that exosomal miRNAs can suppress cancer progression. In pancreatic cancer cells, exosomal miRNA-34a can effectively enter the cell membrane and decrease the expression of Bcl-2 to induce apoptosis and reduce growth and viability. The in vivo experiment on nude mouse xenografts also demonstrated the role of exosomal miRNA-34a in retarding tumor growth [331]. In addition to apoptotic factors, other signaling networks responsible for cancer progression may also be influenced by exosomal miRNAs. It is suggested that exosomal miRNA-210 is a tumor-promoting factor in lung cancer. Secretion of exosomal miRNA-210 by cancer-associated fibroblasts significantly promotes lung cancer migration and invasion. Molecular pathway study reveals that exosomal miRNA-210 induces the PI3K/Akt axis via PTEN down-regulation to induce EMT and enhance lung cancer cell metastasis [332]. UbiA prenyltransferase domain-containing protein 1 (UBIAD1) is downregulated by exosomal miRNA-4644 via binding to its 3’-UTR to enhance bladder cancer cell invasion [333]. Thus, exosomal miRNAs influence a variety of molecular pathways in regulating cancer progression [334–337].

## Exosomal long noncoding RNAs

Recently, lncRNAs have attracted considerable attention because of their potential role in modulating of various molecular signaling pathways in cancer therapy [338–341]. Briefly, lncRNAs are RNA molecules longer than 200 nucleotides and their function differs depending on their localization in the nucleus or cytoplasm [342, 343]. There are five types of lncRNAs and they are able to affect proteins and genes under physiological and pathological conditions [93, 344–347]. The lncRNA DILA1 functions as a tumor-promoting factor and increases the stability of cyclin D1 to promote breast cancer progression and mediate resistance to tamoxifen [348]. Upregulation of lncRNA ENO1-IT1 by the gut microbiota mediates glycolysis and increases the proliferation rate of colorectal cancer cells [349]. Similar to miRNAs, lncRNAs may function as diagnostic and prognostic tools in cancer [350].

Similar to miRNAs, lncRNAs can also be transferred between cells via exosomes. Depending on the function of lncRNAs, they can reduce or promote cancer progression. The lncRNA H19 is considered a tumor-promoting factor because its upregulation induces drug resistance and promote both proliferation and invasion of cancer cells [351]. Exosomes transfer lncRNA H19 to non-small cell lung cancer cells to inhibit apoptosis and induce resistance to gefitinib chemotherapy [352]. Cancer cell migration is also regulated by exosomal lncRNAs. The lncRNA linc-ROR can be transferred into the TME to promote distant metastasis through EMT induction [353]. The exosomal lncRNAs are able to modulate the expression level of miRNAs to target other molecular pathways. The exosomal lncRNA CASC15 is overexpressed in osteosarcomas and increases growth and metastasis. Silencing of CASC15 impairs progression of osteosarcoma cells. Further studies show that exosomal CASC15 decreased the expression of miRNA-338-3p by sponging and increases the expression of RAB14 in osteosarcomas [354]. Delivery of lncRNAs through exosomes is a challenge for the treatment of some kinds of tumor types, particularly brain tumors. The blood–brain barrier (BBB) is an obstacle that prevents antitumor drugs from entering the brain and limits our ability to target brain tumors [355]. However, exosomes are able to disrupt BBB when transporting lncRNAs into the brain. A recent experiment has shown that exosomes are capable of crossing the BBB and transport the lncRNA GS1-6000G8.5 into the brain and mediate metastasis of breast cancer cells to the brain [356].

Due to the interaction between lncRNAs and miRNAs, downregulation of tumor-promoting lncRNAs may pave the way for upregulation of miRNAs with anti-tumor activity. It has been reported that downregulation of the exosomal lncRNA SBF2-AS1 in polarized M2 macrophages leads to the expression of miRNA-122-5p, a tumor suppressor factor. Subsequently, upregulated miRNA-122-5p suppresses pancreatic cancer progression via inhibition of XIAP [357]. As for the ability of exosomal lncRNAs to regulate apoptosis pathways, they can modulate the therapeutic response of cancer cells. The exosomal lncRNA UCA1 shows overexpression in breast cancer cells (MCF-7) and suppresses apoptosis via downregulation of caspase-3 to mediate tamoxifen resistance [358]. In addition to chemotherapy, exosomal lncRNAs regulate the response of cancer cells to radiotherapy. Because of the tumor-promoting role of the lncRNA HOTAIR, its transfer to laryngeal cancer cells via exosomes induces the expression of E2F2 via downregulation of miRNA-454-3p. This accelerates the progression of laryngeal cancer and reduces their sensitivity to radiotherapy [359]. Therefore, the identification of exosomal lncRNAs may increase our understanding of the factors involved in cancer progression and develop novel therapeutics in the near future [360–363].

## Exosomal circular RNAs

CircRNAs are another subset of noncoding RNAs that have a covalently closed loop structure and exhibit vital functions under physiological and pathological conditions [364–367]. Aberrant expression of circRNAs is observed in various cancers. The hsa-circRNA-000166 increases the progression of colorectal cancer by downregulating miRNA-326 and subsequently overexpressing LASP1 [368]. Downregulation of miRNA-665 by circ-100876 occurs in gastric cancer, which triggers EMT via upregulation of YAP1 [369]. Experiments have shown that circRNAs affect cancer growth and metastasis mainly by regulating the expression of miRNAs [369, 370].

The circRNA IARS (circ-IARS) is thought to promote cancer metastasis. This circRNA is located in exosomes and enters HUVECs to increase cancer metastasis. Exosomal circ-IARS decreases overall survival and increases metastasis and TNM stage. Mechanistically, exosomal circ-IARS decreases the levels of miRNA-122 and ZO-1, whereas it increases the levels of RhoA and RhoA-GTP and increases the permeability of endothelial monolayers. Moreover, exosomal circ-IARS enhances F-actin expression and focal adhesion to promote invasion and metastasis [371]. The Wnt signaling pathway is related to cancer proliferation and metastasis. In the context of Wnt pathway, β-catenin translocates to the nucleus to promote cancer progression [372]. The exosomal circ-ABCC1 is overexpressed in colorectal cancer and promotes stemness and invasion. Mechanistically, circ-ABCC1 induces β-catenin to enhance colorectal cancer progression [373]. Similar to lncRNAs, exosomal circRNAs can regulate the response of cancer cells to chemotherapy. Exosomal circ-0002130 shows overexpression in lung cancer and mediates osimertinib resistance. To this end, exosomal circ-0002130 reduces miRNA-498 expression via sponging to enhance GLUT1, HK2, and LDHA expression, leading to lung cancer progression and drug resistance [374]. Another experiment shows how exosomal circRNAs can regulate drug sensitivity via affecting HK2. A recent experiment has shown that exosomal circ-0008928 can increase lung cancer progression and glycolysis. Indeed, exosomal circ-0008928 increases the proliferation rate of lung cancer cells via inducing glycolysis and then, decreases their sensitivity to cisplatin. Molecular pathway study shows that exosomal circ-0008928 enhances HK2 expression in triggering glycolysis and mediating drug resistance in lung cancer [375].

**Fig. 6:**
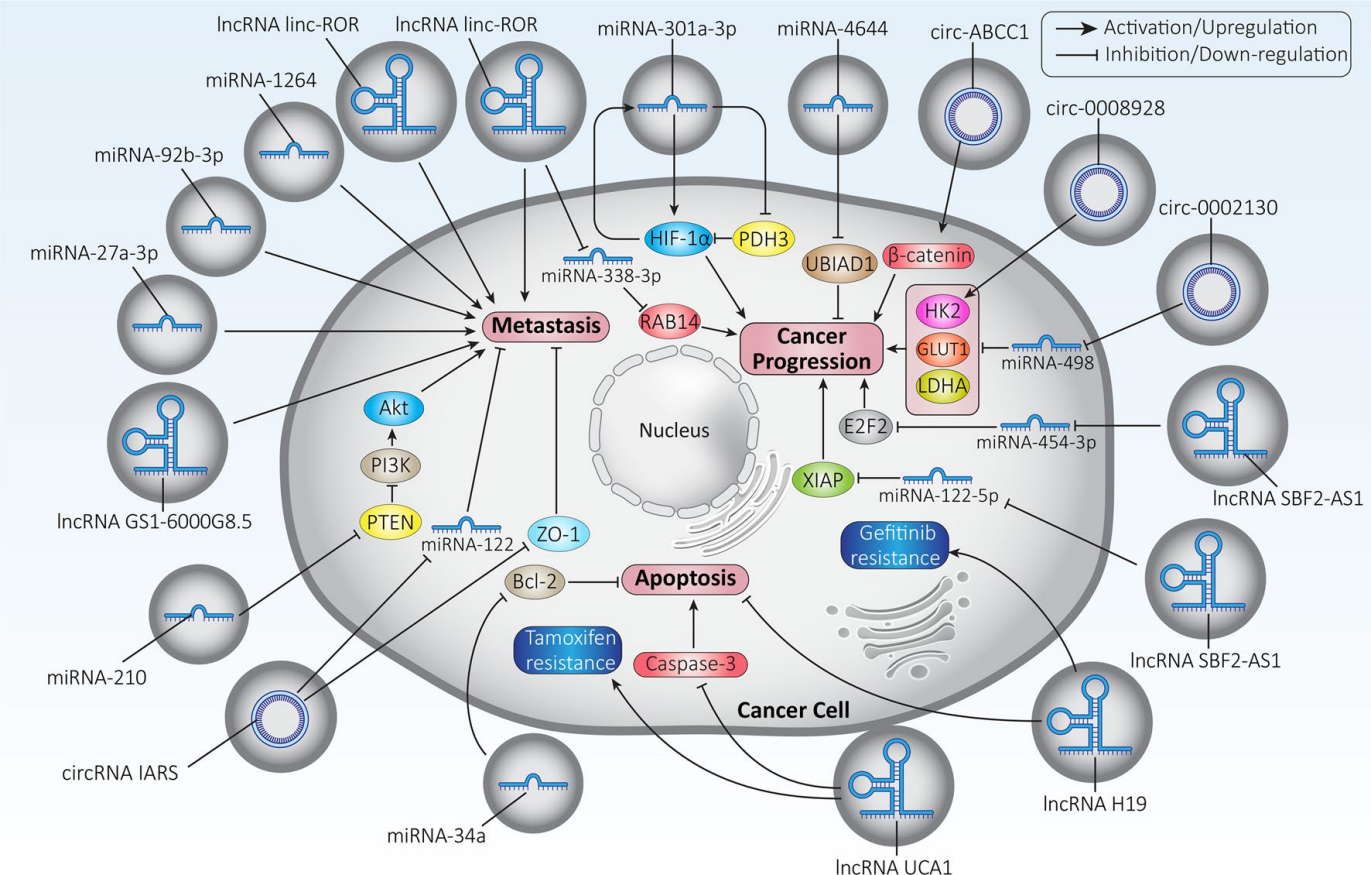
The exosomal ncRNAs in modulating cancer progression. A variety of signaling networks are influenced by exosomal ncRNAs. Metastasis, growth, apoptosis and response to therapy are strongly modulated by exosomal ncRNAs. Further experiments are needed to identify other exosomal circRNAs, as studies have focused more on exosomal miRNAs and lncRNAs

Exosomal circRNAs can also be considered as diagnostic and prognostic tools. For example, the expression levels of circ_0047921, circ_0056285, and circ_0007761 can be used to diagnose non-small cell lung cancer in Chinese. In addition, circ-0056285 is positively associated with the clinical stage and may increase lymph node metastasis [376]. The potential of exosomes as diagnostic and prognostic tools will be specifically discussed in the next sections. However, exosomal circRNAs can be used independently in this case [377–380]. All in all, exosomal ncRNAs regulate proliferation, invasion, immune response and drug sensitivity of cancer cells and can be considered as diagnostic and prognostic factors in cancer (Fig. 6, Table 3) [381–389].

**Table 3:** The exosomal ncRNAs in cancer cells

Exosomal ncRNA	Signaling network	Cancer type	Remarks	Refs
miRNA-34a	–	Breast cancer	Proliferation of cancer cells is suppressed	[390]
miRNA-145	MMP-9 TP53	Breast cancer	Apoptosis is induced Metastasis is inhibited	[391]
miRNA-21-5p	ZNF367	Breast cancer	Cancer cell invasion is suppressed by downregulation of ZNF367	[392]
miRNA-5100	CXC12/CXCR4/EMT	Breast cancer	CXC12/CXCR4 axis is suppressed by miRNA-5100, which acts as a tumor suppressor EMT is inhibited, and cancer cell invasion and migration are decreased	[393]
miRNA-3613-3p	SOCS2	Breast cancer	Cancer cell proliferation and metastasis are enhanced SOCS2 is downregulated	[394]
miRNA-423-5p	–	Breast cancer	The sensitivity of breast cancer cells to cisplatin is reduced	[395]
miRNA-19b-3p	PTEN/EMT	Esophageal cancer	miRNA-19b-3p is upregulated EMT is induced by exosomal miRNA-19b-3p by downregulating PTEN Apoptosis is inhibited Growth and metastasis of cancer cells are enhanced	[396]
miRNA-124	EZH2	Pancreatic cancer	Exosomal miRNA-124 is downregulated Apoptosis is induced, EMT is inhibited and cancer cell migration is decreased by miRNA-124 EZH2 is downregulated by miRNA-124	[397]
miRNA-21-5p miRNA-155-5p	BRG1	Colon cancer	miRNA-21-5p and miRNA-155-5p are transferred from exosomes BRG1 expression is reduced M2 polarization of cancer cells is induced Cancer metastasis is enhanced	[398]
miRNA-34c-3p	Integrin α2β1	Non-small cell lung cancer	Metastasis and invasion of A549 cells are promoted by increased expression of integrins	[399]
miRNA-7	YAP	Lung cancer	YAP expression is inhibited and cancer cell sensitivity to gefitinib is increased	[400]
miRNA-126a	–	Lung cancer	Secretion of exosomes by lung cancer cells is induced by exposure to doxorubicin Cancer cell migration and invasion are increased by exosomal miRNA-126a	[401]
miRNA-122	–	Hepatocellular carcinoma	Sensitivity of cancer cells to chemotherapy is increased by exosomal miRNA-122	[402]
miRNA-302b	ERK1/2 MMP-9 TGFβRII	Lung cancer	Cancer cell growth and invasion are inhibited ERK1/2, MMP-9, and TGFβRII are downregulated	[403]
miRNA-21	PDCD4	Lung cancer	Lung cancer proliferation is increased Anti-tumor immunity is suppressed by the proliferation of myeloid-derived suppressor cells PDCD4 is downregulated	[404]
miRNA-375	ENAH	Esophageal cancer	Cancer progression is suppressed by decreasing the expression of ENAH	[405]
miRNA-146b miRNA-222	–	Papillary thyroid cancer	Proliferation of cancer cells is increased	[406]
miRNA-200b	KLF6	Ovarian cancer	KLF6 is downregulated by miRNA-200b M2 polarization of macrophages is induced	[407]
miRNA-92b-3p	SOX4	Ovarian cancer	Cancer progression is suppressed by inhibiting angiogenesis SOX4 is downregulated	[408]
miRNA-224-5p	–	Renal cancer	Invasion and growth of cancer cells are suppressed	[407]
miRNA-1228	MMP-14	Gastric cancer	Cancer progression is suppressed by downregulation of MMP-14	[408]
lncRNA ZFAS1	Gastric cancer	–	Association with lymph node metastasis and TNM stage is observed EMT is induced Apoptosis is inhibited	[409]
lncRNA KCNQ1OT1	Colorectal cancer	miRNA-30a-5p/USP22/PD-L1	Immune evasion is induced CD8 + T cell response is suppressed Expression of miRNA-30a-5p is decreased by acting as ceRNA USPP22 expression is upregulated to prevent PD-L1 ubiquitination PD-L1 expression is enhanced	[410]
lncRNA HOTAIR	Breast cancer	ErB2	A positive association is observed between HOTAIR and ErB2 HOTAIR expression is increased by ErB2 in a MAPK-dependent manner	[411]
LINC01133	Bladder cancer	Wnt	Low levels of LINC01133 in exosomes from bladder cancer cells are observed Wnt signaling is suppressed to impair cancer cell growth and metastasis	[412]
Circ-ABCC1	Colorectal cancer	Wnt/β-catenin	Cancer cell progression is enhanced by circ-ABCC1 via induction of β-catenin signaling	[376]
Circ-0002130	Non-small cell lung cancer	miRNA-498/HK2-GLUT1-LDHA	Cancer cell proliferation and invasion are increased in vitro and in vivo Osimertinib resistance is observed miRNA-498 is downregulated via sponging Expression of HK2, GLUT1, and LDHA is increased	[374]
Circ-0008928	Non-small cell lung cancer	miRNA-488/HK2	Glycolysis, proliferation and cisplatin resistance of cancer cells are induced Expression of miRNA-488 is decreased to induce HK2 expression	(375)

## Exosomes as carrier systems

### Anti-tumor agents

The previous sections have obviously shown that exosomes can affect cancer progression in several ways and are able to modulate the TME. These effects are based on exosome cargo. In this section, we discuss how exosomes can be used to deliver anti-tumor agents in cancer therapy. Remarkably, exosomes can deliver both synthetic and natural agents. In a recent experiment, exosomes with triptolide were used in the treatment of ovarian cancer. The exosomes showed high encapsulation efficiency and were able to slow tumor growth in vivo. Triptolide-loaded exosomes induce apoptosis in ovarian cancer cells and suppress their proliferation and viability [413]. Paclitaxel (PTX) is an anticancer agent that arrests the cell cycle by disrupting microtubule polymerization. Some cancer cells have developed resistance to PTX chemotherapy. Various techniques including nanoscale delivery systems have been developed to suppress chemoresistance. In one study, exosomes were used as delivery vehicles for PTX in lung cancer therapy. Exosomes were derived from macrophages and then modified with aminoethylanisamide-polyethylene glycol (AA-PEG) to selectively target sigma receptors that are upregulated on the surface of lung cancer cells. These exosomes are preferentially internalized into lung cancer cells and release PTX to suppress lung cancer cell progression [414]. One of the advantages of exosomes is their biocompatibility. In addition, they can deliver drugs as well as act as and imaging agents, which is referred to as theranostics. In a recent experiment, exosomes were isolated from cancer cells (e.g., HeLa cells) and then loaded with doxorubicin. In addition, silver nanoclusters were loaded into doxorubicin-coated exosomes. These exosomes enable imaging while delivering doxorubicin to suppress cancer progression, while exhibiting high biocompatibility and safety profile [415]. In the same study, exosomes were also used to deliver geldanamycin as an HSP90 inhibitor to affect the growth rate of cancer cells [416]. Drug-loaded exosomes can also regulate the TME in favor of cancer therapy. It has been reported that exosomes derived from M1-polarized macrophages can be loaded with PTX. PTX-loaded exosomes induced a pro-inflammatory environment and enhanced inflammation, which promoted the upregulation of caspase-3 expression, triggered apoptosis, and the subsequent enhancement of the anti-tumor activity of PTX [417]. Overall, these studies suggest that exosomes are promising candidates for drug delivery. Further experiments should be performed to elucidate their role in drug delivery, their encapsulation efficiency, and how their surface can be modified to increase their selectivity toward cancer cells [418–421].

### Genetic tools

Small interfering RNAs (siRNAs) are double-stranded RNA molecules of up to 25 nucleotides in length. They are produced from mRNA and lncRNAs via the function of the RNase III enzyme Dicer [422]. The actual function of siRNA is achieved it is incorporated into the RNA-induced silencing complex (RISC) to direct the RNAi machinery to target mRNA for degradation after complementary sequences are found. Recently, siRNA has paved the way to treat various diseases in preclinical and clinical research, such as viral infections, neurological disorders, ocular diseases, autoimmune diseases, and cancer [421, 423]. Although siRNA has shown great capacity in suppressing gene expression and subsequently treating disease, naked siRNA appears to require modification in alleviating disease. Degradation of siRNA by RNase enzymes, tumor barriers, and off-targeting are drawbacks of siRNA that can be solved using delivery systems [424, 425]. Another genetic tool used in cancer therapy is the CRISPR/Cas system. The CRISPR/Cas9 system is the best known type of CRISPR system that has recently been used in the treatment of diseases [426]. The CRISPR/Cas9 system was discovered in prokaryotes and its main function is adaptive immunity [427]. The CRISPR/Cas9 system consists of three main components, including Cas9, sgRNA, and tracrRNA. The specificity, efficiency, and accuracy of the CRISPR/Cas9 system are provided by sgRNA. Cas9 acts as a scissor and is responsible for the destruction of double-stranded DNA [428]. Various experiments have been conducted on the use of CRISPR/Cas9 system in cancer therapy. CRISPR/Cas9 is able to target fusion oncogenes or transcription factors to suppress cancer progression and reduce growth and mortality [429]. Downregulation of ZEB1 and ZEB2 by the CRISPR/Cas9 system significantly reduces lung cancer cell migration and invasion [430]. The present section addresses the role of exosomes in the delivery of siRNA and the CRISPR/Cas system in cancer therapy.

#### siRNA

In one experiment, exosomes were isolated from embryonic kidney cells (HEK-293 cells) by ultracentrifugation and loaded with siRNA. The resulting exosomes had a diameter of 107 nm and an encapsulation efficiency of 10–20%. The exosomes efficiently transported siRNA to PANC-1 cells, demonstrating their potential as a transport system [431]. Induction of apoptosis is an ideal strategy to suppress cancer proliferation. To this end, Bcl-2-siRNA was loaded into exosomes and its anti-tumor activity against digestive system tumors was evaluated. Bcl-2-siRNA-loaded exosomes penetrated the cell membrane and delivered siRNA, resulting in apoptosis induction and reduced tumor growth. The anti-tumor activity of these exosomes was confirmed in both in vitro and in vivo experiments [432].

Hepatocyte growth factor (HGF) was first identified in mouse liver and is considered a cytokine with physiological functions in cell proliferation, survival, and migration [433, 434]. Recent experiments have revealed the tumor-promoting role of HGF in cancer. Overexpression of HGF enhances the growth and invasion of cervical cancer cells via affecting c-Met [435]. By inducing the c-Met/PI3K/Akt axis, HGF induces EMT and mediates drug resistance in pancreatic cancer [436]. HGF-siRNA-loaded exosomes may serve as nanostructures for cargo transport in gastric cancer treatment. These exosomes effectively transport siRNA to gastric cancer cells, leading to a significant reduction in their growth and migration and inhibition of angiogenesis [437]. Polo-like kinase 1 (PLK1) is another tumor-promoting factor in cancer. Overexpression of PLK1 inhibits autophagic cell death in prostate cancer [438]. In addition, Silencing of PLK1 suppresses breast cancer cell migration and invasion and promotes their sensitivity to drugs [439]. PLK1-siRNA was introduced into exosomes by electroporation, and exposure of bladder cancer cells to these exosomes resulted in a significant decrease in PLK1 mRNA levels and subsequent cancer eradication [440].

Efforts are underway to engineer exosomes to increase their selectivity toward cancer cells. One of the promising methods is to modify exosomes with ligands. The exosomes carrying DARPin G3 on their surface can bind to HER2/Neu on breast cancer cells. These targeted exosomes deliver TPD52-siRNA and reduce the expression of HER2/Neu by up to 70% [441]. Future experiments may therefore focus on the development of engineered and surface-modified exosomes for cancer therapy. Another ligand that can be used for surface modification of exosomes is the tLyp-1 peptide with the amino acid sequence CGNKRTR. The tLyp-1 peptide is able to bind to receptors such as neuropilin-1 (NRP1) and neuropilin-2 (NRP2), which are overexpressed on the surface of lung cancer cells [442–444]. Recently, tLyp-1 exosomes were used for siRNA delivery. These nanostructures were 100 nm in diameter and, thanks due to their selectivity toward lung cancer cells, they efficiently delivered siRNA and significantly reduced cancer stemness [445].

The factors involved in cancer metabolism and growth can be targeted by siRNA to increase drug sensitivity. Recently, CPT1A-siRNA was loaded into exosomes and surface modification with iRGD was performed to promote their selectivity toward colon cancer cells. These exosomes increased the sensitivity of colon cancer cells to oxaliplatin by downregulating CPT1A. Simultaneous administration of these exosomes and oxaliplatin induced apoptosis in colon cancer cells and suppressed their proliferation [446]. In another experiment, exosomes were modified with an epidermal growth factor receptor (EGFR) aptamer and then loaded with survivin-siRNA. Since survivin is involved in cancer progression and functions as an anti-apoptotic factor, its downregulation by exosomes sensitizes lung cancer cells to apoptosis. Exosomes provide endosomal escape of siRNA, which is important for increasing its efficiency in anticancer activity [447]. Overall, it is evident that siRNA is an efficient tool in cancer therapy and exosomes enhance its potential to suppress cancer and promote chemosensitivity [448]. Short hairpin RNA (shRNA) is another genetic tool that has a similar function to siRNA and can be used to regulate gene expression in cancer therapy. However, there is no study evaluating the potential of exosomes in shRNA, and further experiments could focus on this aspect.

#### CRISPR/Cas9 system

**Fig. 7:**
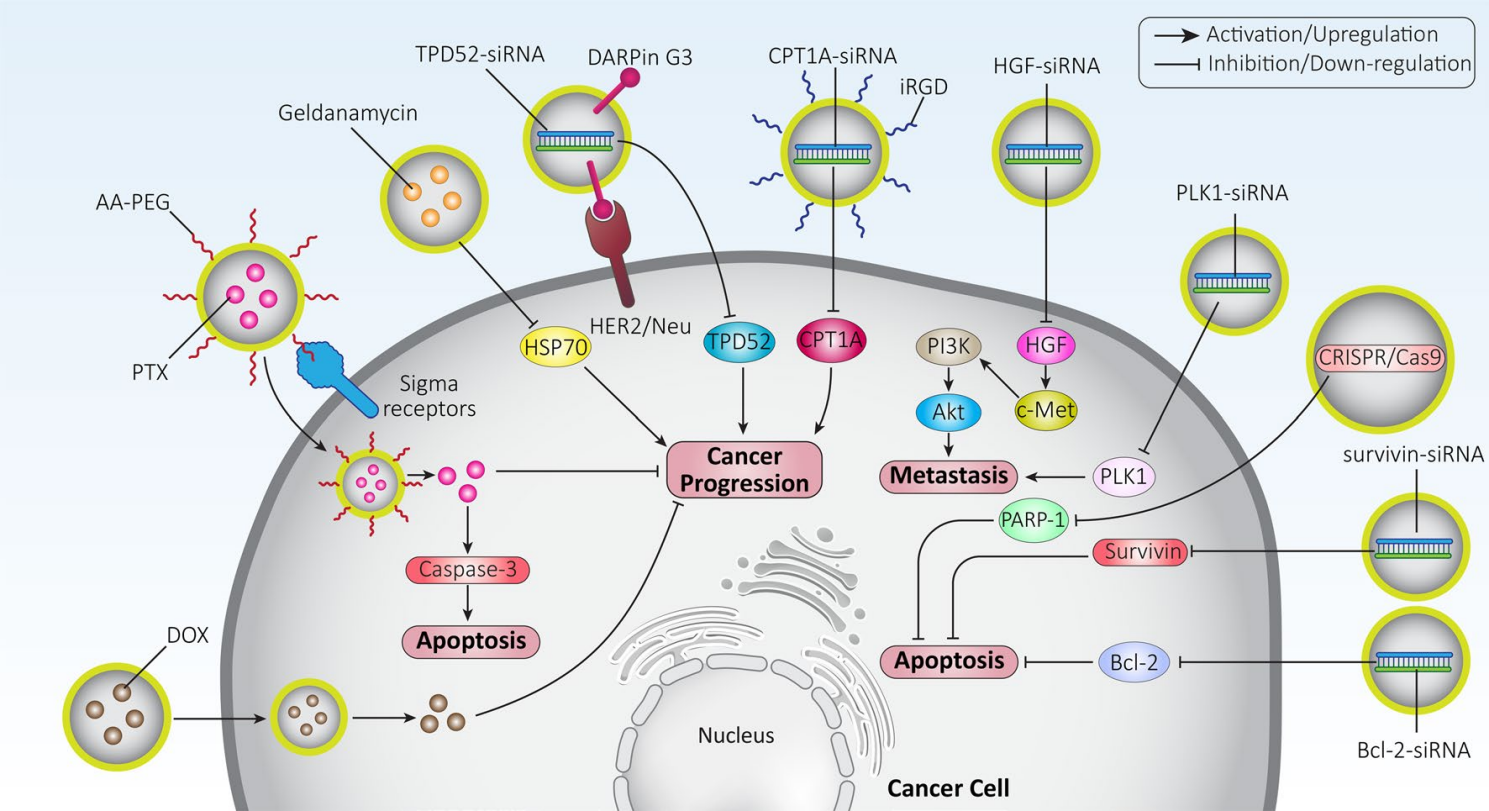
The use of exosomes in the administration of genetic tools. Downregulation of tumor-promoting molecular signaling pathways such as survivin, Bcl-2, PLK1, HGF, and TPD52 by exosomes loaded with genetic tools leads to induction of apoptosis, impairment of tumor progression, and suppression of cancer metastasis

To date, only two studies have focused on CRISPR/Cas9 transfer through exosomes. In one experiment, cancer-derived exosomes were used to transfer the CRISPR/Cas9 system in the treatment of ovarian cancer. It seems that cancer-derived exosomes have high selectivity toward ovarian cancer cells due to cell tropism. CRISPR/Cas9-loaded exosomes significantly stimulated apoptosis by downregulating PARP-1. In addition, CRISPR/Cas9-loaded exosomes are able to increase the cytotoxicity of cisplatin (CP) against ovarian cancer cells [449]. Limitations of the CRISPR/Cas9 system include its difficulty in specifically targeting all cancer cells and its low efficacy in vivo. Therefore, it seems crucial to use exosomes for the delivery of the CRISPR/Cas9 system in cancer therapy. Exosomes can induce necroptosis in cancer cells through the CRISPR/Cas9 system (Fig. 7) [450]. However, few studies have investigated this potential of exosomes, and the development of engineered and surface-modified exosomes is encouraged.

## Tumor-derived exosomes

### Brain tumors

The exosomes can be derived from glioblastoma cells. These exosomes mediate immune evasion of cancer cells via induction of PD-L1 expression and transfer of STAT3. In addition, exosomes induce M2 polarization of macrophages, which promotes glioblastoma progression [451]. When glioblastoma cells are exposed to hypoxia, the secretion of exosomes is triggered. These exosomes contain miRNA-1246, which is a tumor-promoting factor to promote cancer progression by upregulating STAT3 expression, suppressing NF-κB signaling, and mediating M2 polarization of macrophages [452]. Glioblastoma-derived exosomes contain high levels of VEGF-C, which inhibits Hippo signaling and enhances tafazzin (TAZ) expression, leading to angiogenesis [453]. Proteomics can be used to identify proteins embedded in exosomes and use them as biomarkers [454]. It appears that glioblastoma-derived exosomes have immunomodulatory effects. As mentioned previously, these exosomes are able to mediate M2 polarization of macrophages, which is attributed to the induction of NF-κB signaling. Then, macrophages secrete factors responsible for cancer progression. Moreover, these exosomes suppress the activity of cytotoxic CD4 + T cells to evade the immune response in glioblastomas [455]. Furthermore, exosomes from glioblastomas promote cancer stemness by transferring Notch1 protein [456].

A recent experiment has shown that exosomes derived from glioma stem cells contain high levels of Linc01060, which acts as a tumor-promoting factor and promotes cancer progression. The exosomal Linc01060 increases the stability of myeloid zinc finger 1 (MZF1) as a transcription factor and induces its nuclear translocation. Then, MZF1 induces HIF-1α via upregulation of c-Myc to enhance glioma progression [457]. Glioma-derived exosomes contain high levels of miRNA-10a and miRNA-21, which regulate PTEN and RORA, leading to activation of myeloid-derived suppressor cells and impairing immune function [458]. miRNA-1246 and mIRNA-10b-5p are other miRNAs found in glioma-derived exosomes that promote cancer cell metastasis [459]. Furthermore, glioma cells secrete exosomes containing the lncRNA CCAT2 to stimulate angiogenesis and inhibit apoptosis, setting the stage for tumor progression [460]. To trigger angiogenesis, glioma-derived exosomes may deliver miRNA-21, which upregulates VEGF expression [461]. Overall, these studies are consistent with the fact that glioma-derived exosomes modulate proliferation and migration by transporting various cargoes [462–464].

### Thoracic and breast tumors

Breast cancer-derived exosomes are capable of suppressing immune function to enhance tumor progression. Injection of exosomes into naïve mice leads to accumulation of myeloid-derived suppressor cells in the lungs and the liver. Breast cancer-derived exosomes prevent T cell proliferation and suppress natural killer cell cytotoxicity to mediate immune evasion [465]. In enhancing cancer progression, breast cancer-derived exosomes transfer miRNA-155 to induce cachexia via downregulation of PARP-1 expression. Further studies revealed that these exosomes can also induce EMT-mediated metastasis in breast cancer by triggering catabolism and release of metabolites in adipocytes and muscle cells [466]. Metastatic breast cancer cells secrete exosomes containing miRNA-21 and miRNA-200c, which can be detected in patients and are used as diagnostic and prognostic factors [467]. The protein content of exosomes can be analyzed to distinguish breast cancer subtypes [468]. The presence of CD44 in breast cancer-derived exosomes leads to doxorubicin resistance [469]. Moreover, activation of fibroblasts by exosomes containing survivin can promote both growth and metastasis of breast cancer cells [470]. By inducing M2 polarization of macrophages, breast cancer-derived exosomes enhance lymph node metastasis of breast cancer cells [471]. Therefore, breast cancer-derived exosomes may modulate the progression of these tumor cells [472].

Exosomes derived from lung cancer cells, on the other hand, may act as triggers of EMT via upregulation of vimentin [473]. Exosomes derived from gemcitabine-resistant cancer cells may promote the progression of non-small cell lung cancer cells, mediate their drug resistance, and enhance their malignant phenotype through the transmission of miRNA-222-3p [474]. Exosomes derived from lung cancer cells are able to induce the Wnt3a/β-catenin axis to promote growth and survival [475]. Irradiation stimulates the release of exosomes from non-small cell lung cancer and induces Akt, STAT3, and ERK signaling pathways that mediate resistance to kinase inhibitors [476]. In additions, some of the proteins, such as MUC1, are enriched in exosomes to determine their localization and biological function [477].

### Gastrointestinal tumors

Exosomes derived from gastric cancer enhance peritoneal metastasis and disrupt the mesothelial barrier [478]. These exosomes induce NF-κB signaling in macrophages to mediate secretion of pro-inflammatory factors and promote gastric cancer progression [479]. Induction of NF-κB signaling by gastric cancer-derived exosomes maintains inflammatory conditions in the TME that promote gastric cancer progression [480]. Gastric cancer-derived exosomes are able to stimulate PI3K/Akt and MERK/ERK signaling pathways. Moreover, inhibition of BMP prevents the potential of exosomes to transform pericytes into cancer-associated fibroblasts [481]. Exposure of gastric cancer cells to various antitumor agents may affect their ability to secrete exosomes. Pyrotinib, for example, induces the release of exosomes from gastric cancer cells. The secreted exosomes enhance migration, and the use of apatinib as a VEGFR inhibitor, suppresses this condition [482].

Hepatocellular carcinoma is another gastrointestinal tumor. Exosomes derived from hepatocellular carcinoma cells can induce ERK signaling to mediate EMT via upregulation of ZEB1/2, leading to cancer metastasis [483]. In addition to metastasis, exosomes derived from hepatocellular carcinomas also mediate cancer recurrence and can be used for early diagnosis of this malignancy [484]. By triggering chaperone-mediated autophagy, hepatocellular carcinoma-derived exosomes induce drug resistance and inhibit apoptosis [485]. Exosomes are also known to transfer Linc-ROR to liver cancer cells to increase their growth rate and inhibit their apoptosis [485].

Pancreatic cancer cells may also secrete exosomes. A recent experiment has shown that Dickkopf1 (DKK1)-dependent endocytosis is involved in the biogenesis of exosomes. Pancreatic cancer cell-derived exosomes have high levels of CKAP4 and are associated with poor prognosis in patients [486]. Moreover, pancreatic cancer-derived exosomes mediate M2 polarization of macrophages to suppress immune function against cancer cells [487]. To demonstrate the potential of exosomes in cancer migration, an experiment isolated serum exosomes from pancreatic cancer patients and showed that they can induce EMT and promote metastasis [488]. To enhance invasion and migration ability, pancreatic cancer cell-derived exosomes recruit cancer-associated fibroblasts and transfer Lin28B to reduce let-7 expression, leading to upregulation of HMGA2 and subsequent overexpression of PDGFB [489]. All in all, exosomes derived from pancreatic cancer cells regulate cancer progression, and their isolation and targeting may be important for cancer therapy [489–494].

### Reproductive tumors

**Fig. 8:**
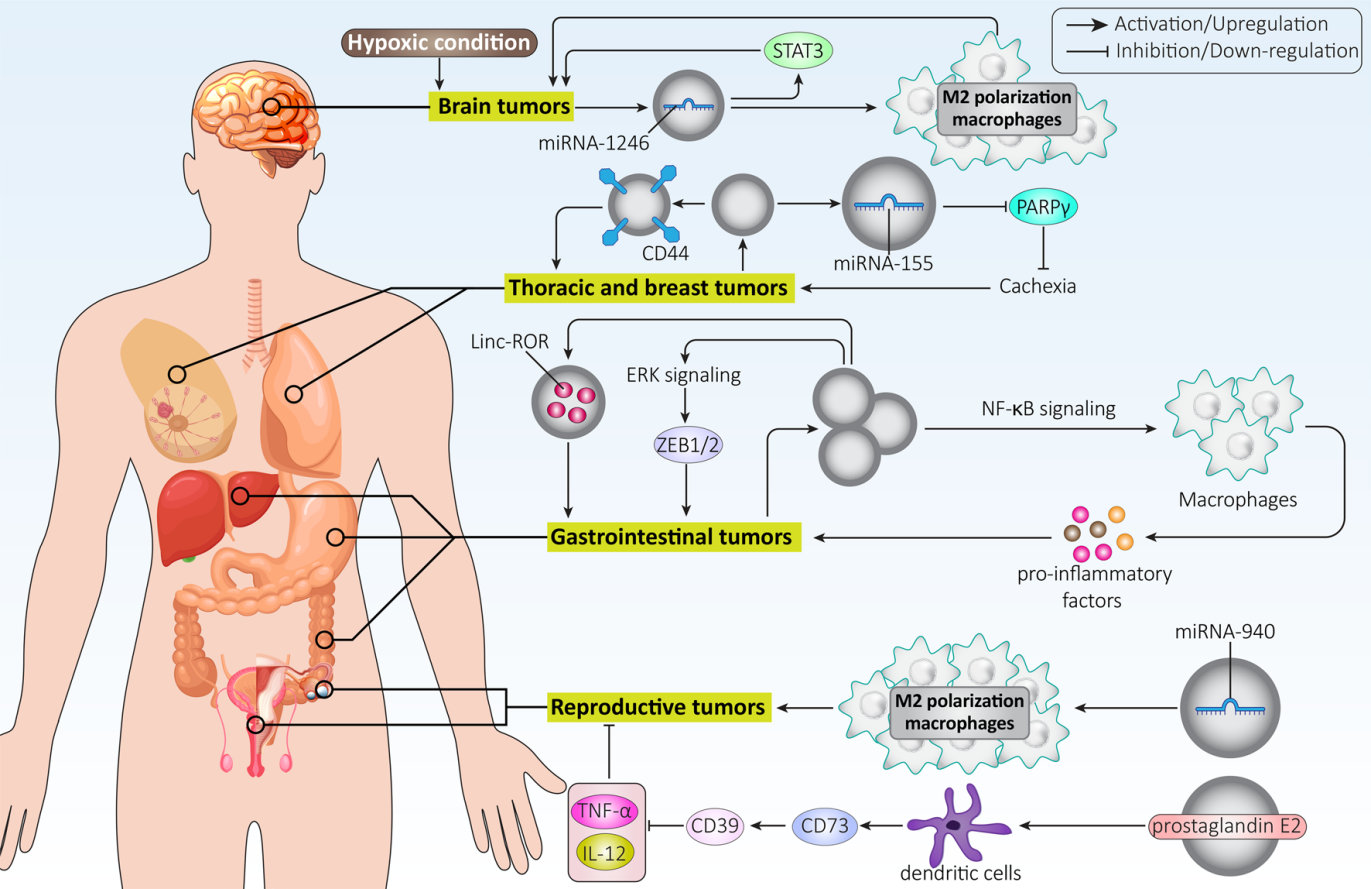
Tumor-derived exosomes and their role in cancer progression

Most experiments on reproductive tumor-derived exosomes have focused on ovarian cancer. Proteomic and lipidomic analysis of exosomes can be used in the early diagnosis of ovarian cancer [495]. Ovarian cancer cell-derived exosomes may be involved in the development of malignant TME by promoting fibroblast migration [496]. They can be considered as potential therapeutic targets, as their modulation can suppress growth and invasion of ovarian cancer cells [497]. Moreover, ovarian cancer cell-derived exosomes can transport miRNAs into the TME and induce M2 polarization of macrophages that promote cancer progression [498]. Exosomal miRNA-940 stimulates ovarian cancer progression by inducing polarization of macrophages to the M2 phenotype [499]. Ovarian cancer-cell derived exosomes can induce angiogenesis and migration via upregulation of VEGF [500]. The same phenomenon occurs in cervical cancer. It has been reported that cervical cancer cell-derived exosomes can promote angiogenesis via Hedgehog-GLI signaling and enhancement of VEGF-A, VEGFR2, and angiopoietin-2 expression [501]. Loading dendritic cells with exosomes derived from HeLa cells stimulates anti-tumor immunity by increasing T-cell cytotoxicity [502]. Overall, these experiments highlight the role of ovarian and cervical cancer cell-derived exosomes in modulating migration, growth, TME, anti-tumor immunity, and angiogenesis. An experiment was conducted to investigate the role of prostate cancer-derived exosomes in immunomodulation. These exosomes impair dendritic cell function and suppress CD8 + T cell activity. The exosomes mediate the expression of CD73 on dendritic cells, which subsequently upregulate the expression of CD39, resulting in ATP-dependent inhibition of TNF-α and IL-12 production. In addition, exosomes have been found to contain prostaglandin E2, which enhances CD73 expression (Fig. 8) [503].

## Clinical application and role of exosomes as biomarkers

According to the role of exosomes in influencing cancer progression, a significant attempt has been made to reveal the role of exosomes in clinical background [504–508]. The genes contained in exosomes can be considered as biomarkers for the diagnosis of lung cancer. A recent experiment has shown that exosomes derived from non-small cell lung cancer cells have Hippo, Rap1, and Wnt as important signaling networks and can be considered as prognostic tools [509]. Another study shows that exosomes derived from non-small cell lung cancer contain high levels of PRPS2 and can mediate cisplatin resistance. In addition, PRPS2-containing exosomes are capable of inducing M2 polarization of macrophages and are associated with an unfavorable prognosis [510]. Exosomes containing high levels of miRNA-3362, miRNA-146a, and miRNA-1290 are observed in breast cancer patients and mediate lymph node metastasis and clinical stage [511]. In colorectal cancer patients, the abundance of QSOX1 in plasma exosomes decreases and can be considered as a diagnostic factor [511]. These studies demonstrate how exosomes can be analyzed to determine the prognosis of cancer patients (Table 4).

**Table 4:** Clinical trials on the use of exosomes in cancer patients

Status	Remarks	Reference
Completed	Use of exosomes as reliable biomarkers for the diagnosis of men with prostate cancer	NCT02702856
Unknown	Combination of computed tomography and exosomes for diagnosis of early stage lung cancer	NCT03542253
Active, not recruiting	Use of exosomes present in blood plasma to diagnose lung cancer in patients	NCT04529915
Unknown	Use of circulating exosomes for diagnosis of advanced gastric cancer	NCT01779583
Recruiting	New diagnostic method for colorectal cancer using exosomes	NCT04394572
Recruiting	Presence of exosomes in tumor-draining vein and their molecular profiling	NCT04939324
Recruiting	Use of plant exosome for delivery of curcumin in the treatment of colon cancer	NCT01294072

## Conclusion and future perspectives

Thanks to the attempts made in recent years to uncover the factors involved in cancer progression, it is now clear that each factor has a unique fingerprint in cancer pathogenesis. If we know exactly how these factors interact in cancer, we can develop novel and effective therapeutics. Exosomes are minute structures that are involved in the regulation of biological processes through their cargo, which can be proteins, lipids, or nucleic acids. Genetic tools and anti-tumor agents can also be loaded into exosomes. Therefore, they provide intercellular communication and their involvement in cancer progression or inhibition is inevitable. Depending on the cargo, the effect of exosomes on the target cell may be different. In addition, normal cells such as macrophages and mesenchymal stem cells are capable of secreting exosomes to affect cancer cell progression. Therefore, exosomes can mediate both normal cell-cancer cell and cancer cell-cancer cell interactions.

Growth and invasion are the two most important aspects of tumor cells. When exosomes contain tumor-promoting substances, they can promote cell cycle progression and glycolysis and inhibit apoptosis. The role of autophagy is a bit confusing. It has been mentioned that induction of autophagy by exosomes can prevent apoptosis in cancer cells. Therefore, further experiments should be performed to reveal the interaction between apoptosis and autophagy in cancer cells affected by exosomes. Similar to proliferation, the cargo of exosomes determines the function of these structures in increasing or decreasing cancer migration and invasion. EMT and MMPs are strongly influenced by exosomes in regulating cancer progression. However, most studies have focused on the EMT mechanism, and it is proposed to uncover the signaling networks affected by exosomes in targeting MMPs and modulating cancer metastasis, because of the important role of MMPs in this case. Cancer cell proliferation and invasion rates determine response to therapy. If cancer cells have a high capacity to migrate and grow, they may develop resistance to therapy. Therefore, by targeting exosomes, proliferation and invasion of cancer cells can be modulated and their response to therapy can be predicted. The aggressive behavior of cancer cells depends mainly on interactions in the TME. The best known interaction in the TME is macrophage polarization mediated by exosomes. Exosomes can induce M2 polarization of macrophages, promoting cancer cell progression.

Since response to therapy is a major concern for physicians treating cancer patients, we have provided a section specifically addressing the role of exosomes in this case. The sensitivity of cancer cells to chemotherapy-mediated apoptosis can be reduced by exosomes. Because of the potential of exosomes to transfer various genes, they can influence the progression of cancer cells and determine their response to therapy. In addition to drug resistance, exosomes may also be involved in triggering radioresistance. In addition, exosomes can induce immune cell exhaustion, decrease T cell cytotoxicity, and mediate immune evasion. By triggering chronic inflammation, exosomes promote cancer progression. When new therapeutics are to be introduced into clinics for the treatment of cancer patients, they can focus on these aspects.

For internalization into cells, exosomes can follow different pathways. Exosomes are able to bind to receptors on the surface of cells, and can be internalized by binding to integrins, tetraspanins and intercellular adhesion molecules. Clathrin- and caveolin-mediated endocytosis, lipid raft uptake, macropinocytosis, phagocytosis and fusion with the plasma membrane [512]. Therefore, if exosomes are to be used for cargo transport in cancer therapy, the method of their internalization should be elucidated and subsequent functionalization should be performed to improve their intracellular accumulation.

Exosomes may contain miRNAs, lncRNAs, circRNAs, and other genes such as PTEN, PI3K/Akt, and STAT3 that affect cancer progression. Indeed, cancer progression is strongly influenced by exosome cargo. Since exosomes are capable of delivering various drugs, they have the potential to be used as delivery systems for anti-tumor agents and genetic tools in cancer therapy. The various anti-cancer agents, including plant-derived natural compounds such as triptolides and synthetic agents such as cisplatin, doxorubicin and paclitaxel, can be transferred by exosomes in cancer therapy. The siRNA and CRISPR/Cas9 are genetic tools embedded in exosomes for cancer therapy. Delivery of therapeutics using exosomes can potentially lead to increased intracellular accumulation and improved therapeutic efficacy. In addition, the surface of exosomes can be modified with ligands to increase their selectivity toward cancer cells. Since exosomes affect various aspects of cancer cells, they can be isolated from the serum of patients and are considered reliable biomarkers for the diagnosis and prognosis of cancer patients. As shown in Table 4, exosomes have been used as biomarkers in various experiments in cancer patients. In addition to diagnosis, exosomes have also been used to increase the accuracy of other methods of detecting cancer patients, such as CT. Of note, a clinical trial is currently underway to deliver curcumin as an anti-cancer agent to treat colon cancer. The results of this clinical trial are of great importance, as they may provide novel insights into the role of exosomes as drug delivery systems in the clinical course and their safety. Furthermore, a number of clinical trials on molecular profiling of exosomes are currently ongoing, which could be useful in the field of precision medicine in the near future.

## Data Availability

Not applicable.

## Abbreviations

CSC: Cancer stem cell
EVs: Extracellular vesicles
TME: Tumor microenvironment
ncRNAs: Non-coding RNAs
miRNA: MicroRNA
lncRNA: Long non-coding RNA
circRNA: Circular RNA
MVBs: Multi-vesicular bodies
ILVs: Intraluminal vesicles
ESCRT: Endosomal sorting complex required for transport
VTA1: Vesicle trafficking 1
VPS4: Vacuolar protein sorting-associated protein 4
TSG101: Tumor susceptibility gene 101 protein
HSP60: Heat shock protein 60
Rab: Ras-associated binding
SNARE: Soluble NSF-attachment protein receptor
STAT3: Signal transducer and activator of transcription 3
IL-6: Interleukin-6
MMP-9: Matrix metalloproteinase-9
ROS: Reactive oxygen species
BBB: Blood–brain barrier
ERK: Extracellular-signal-regulated kinase
SIRT2: Sirtuin 2
TFEB: Transcription factor EB
EphA2: Eph receptor A2
EMT: Epithelial-to-mesenchymal transition
EMT-TFs: EMT-inducing transcription factors
MMPs: Matrix metalloproteinases
TFF3: Trefoil factor 3
VEGF: Vascular endothelial growth factor
VEGFR2: VEGF receptor 2
NRP-1: Neuropilin-1
OSCC: Oral squamous cell carcinoma
NPS: Nasopharyngeal carcinoma
ANGPT2: Angiopoietin-2
HCC: Hepatocellular carcinoma
HIF-1α: Hypoxia inducible factor-1α
TEX: Tumor-derived exosomes
5-FU: 5-Fluorouracil
CP: Cisplatin
TGF-β: Transforming growth factor-beta
Treg cells: T regulatory cells
PD-1: Programmed death-1
IDO: Indoleamine 2:3-dioxygenase
3^/^-UTR: 3^/^-Untranslated region
PTEN: Phosphatase and tensin homolog
PTX: Paclitaxel
AA-PEG: Aminoethylanisamide-polyethylene glycol
RISC: RNA-induced silencing complex
HGF: Hepatocyte growth factor
PLK1: Polo-like kinase 1
EGFR: Epidermal growth factor receptor
shRNA: Short hairpin RNA
TA2: Tafazzin
MZF1: Myeloid zinc finger 1
DKK1: Dickkopf1
